# Top-down inputs drive neuronal network rewiring and context-enhanced sensory processing in olfaction

**DOI:** 10.1371/journal.pcbi.1006611

**Published:** 2019-01-22

**Authors:** Wayne Adams, James N. Graham, Xuchen Han, Hermann Riecke

**Affiliations:** 1 Engineering Sciences and Applied Mathematics, Northwestern University, Evanston, IL, USA; 2 Mathematics, Northwestern University, Evanston, IL, USA; University of Pittsburgh, UNITED STATES

## Abstract

Much of the computational power of the mammalian brain arises from its extensive top-down projections. To enable neuron-specific information processing these projections have to be precisely targeted. How such a specific connectivity emerges and what functions it supports is still poorly understood. We addressed these questions in silico in the context of the profound structural plasticity of the olfactory system. At the core of this plasticity are the granule cells of the olfactory bulb, which integrate bottom-up sensory inputs and top-down inputs delivered by vast top-down projections from cortical and other brain areas. We developed a biophysically supported computational model for the rewiring of the top-down projections and the intra-bulbar network via adult neurogenesis. The model captures various previous physiological and behavioral observations and makes specific predictions for the cortico-bulbar network connectivity that is learned by odor exposure and environmental contexts. Specifically, it predicts that—after learning—the granule-cell receptive fields with respect to sensory and with respect to cortical inputs are highly correlated. This enables cortical cells that respond to a learned odor to enact disynaptic inhibitory control specifically of bulbar principal cells that respond to that odor. For this the reciprocal nature of the granule cell synapses with the principal cells is essential. Functionally, the model predicts context-enhanced stimulus discrimination in cluttered environments (‘olfactory cocktail parties’) and the ability of the system to adapt to its tasks by rapidly switching between different odor-processing modes. These predictions are experimentally testable. At the same time they provide guidance for future experiments aimed at unraveling the cortico-bulbar connectivity.

## Author summary

In mammalian sensory processing, extensive top-down feedback from higher brain areas reshapes the feedforward, bottom-up information processing. The structure of the top-down connectivity, the mechanisms leading to its specificity, and the functions it supports are still poorly understood. Using computational modeling, we investigated these issues in the olfactory system. There, the granule cells of the olfactory bulb, which is the first brain area to receive sensory input from the nose, are the key players of extensive structural changes to the network through the addition and also the removal of granule cells as well as through the formation and removal of their connections. This structural plasticity allows the system to learn and to adapt its sensory processing to its odor environment. Crucially, the granule cells combine bottom-up sensory input from the nose with top-down input from higher brain areas, including cortex. Our biophysically supported computational model predicts that, after learning, the granule cells enable cortical neurons that respond to a learned odor to gain inhibitory control of principal neurons of the olfactory bulb, specifically of those that respond to the learned odor. Functionally, this allows top-down input to enhance odor discrimination in cluttered environments and to quickly switch between odor tasks.

## Introduction

A key property of the mammalian brain that is essential for its vast computational power is the pervasiveness of centrifugal, top-down feedback from higher to lower brain areas [1–5]. The top-down projections can provide the receiving brain area with information that is not available in the feedforward stream [6] and can switch the processing by the lower brain area between different modes, as demonstrated in the visual system [7]. From a theoretical perspective, it has been posited that top-down signals can direct the lower brain area to suppress response to expected inputs or to inputs that have already been recognized by the higher brain area [8–11] and to transmit predominantly information about unexpected or unexplained inputs and the error in the prediction of the inputs [12], focusing on task-relevant information. Such specificity in the processing requires that the top-down projections be precisely targeted [10, 13–16]. The mechanisms that are at work in the formation of these specific connectivities are, however, not well understood. Experiments in the visual system suggest that they are activity-dependent [17]. Here we address this issue in the context of the extensive structural plasticity of the adult olfactory system.

In the olfactory bulb, which is the first brain area receiving sensory input from the nose, structural plasticity is not restricted to early development, but is also pronounced in adult animals. At that point its key players are the granule cells (GCs). They receive sensory input from the bulb’s principal neurons—mitral and tufted cells (MCs)—and are the target of massive top-down projections from the olfactory cortex [2–5]. Not only do the GC dendritic spines exhibit strong and persistent fluctuations [18, 19], but these interneurons themselves, which constitute the dominant neuronal population of the bulb, undergo persistent turnover through adult neurogenesis [20]. Both, spine fluctuations and the survival of the granule cells depend on the sensory environment [18, 21].

Functionally, adult neurogenesis is observed to enable and improve various aspects of learning and memory [22, 23]. A particularly clear example is the perceptual learning of a spontaneous odor discrimination task, which is substantially compromised if adult neurogenesis is suppressed [24]. The survival of GCs seems also to play an important role in retaining the memory of an odor task, with the survival of odor-specific GCs being contingent on the continued relevance of that odor memory [25]. This suggests that GCs receive non-sensory, task-related information via the top-down projections. These projections likely also underlie the GC activation that can be evoked by context alone, without the presence of any odor, if that context has previously been associated with an odor [26]. Importantly, the context-evoked GC activation patterns reflect specifically the GC activation pattern that would be induced by the associated odor.

Taken together, the experiments suggest that sensory input together with non-olfactory information like context or valence may shape the connectivity within the olfactory bulb as well as that of the top-down projections onto the GCs. It is, however, poorly understood how the bottom-up and top-down inputs into the GCs jointly shape the network connectivity, what mechanisms are at work, what kind of connectivities arise, and what kind of functionalities these connectivities support. To address these issues we have employed computational modeling using a biophysically supported framework. This model makes a number of predictions that can be tested with physiological and behavioral experiments and that can guide future experiments aimed at unraveling the cortico-bulbar connectivity.

## Results

We have developed a computational network model for the olfactory bulb and its bidirectional communication with a cortical area, which is based on key features of adult neurogenesis as observed experimentally. Details of the model are given in Methods. Briefly, the bulbar component of the model network comprised the two main neuronal populations of the olfactory bulb, the mitral/tufted cells (MCs) and the granule cells (GCs). The MCs are known to project from the olfactory bulb to a host of other brain areas, including the anterior olfactory nucleus, the anterior and the posterior piriform cortex, the olfactory tubercle, the lateral entorhinal cortex, and the cortical amygdaloid nucleus [27–29]. Each of these areas presumably extracts different aspects of the olfactory information from the MC activation patterns. Here we are interested in the top-down projections that reach the olfactory bulb from olfactory cortex. Experiments indicate that parts of olfactory cortex perform pattern *completion* [30–32] rather than the pattern *separation* that is observed for the bulbar output [33–35]. This pattern completion is presumably due to excitatory associational fibers. To wit, when presenting multi-odor mixtures the cortical representation of a mixture of 10 odors changed only little when one of the odors was omitted, but changed much more when one odor was replaced [30–32]. This suggests that the cortical network filled in the missing information when one component was omitted. In contrast, in these experiments the bulbar representations changed substantially already when a single odor was omitted from the mixture. Moreover, the pattern completion depended on the training of the animals, reflecting plastic processes [31].

Motivated by the observation of cortical pattern completion we included a minimal model of an associational brain area through a third population of principal, excitatory cells (CCs), which were connected with each other through plastic excitatory connections as well as fixed inhibitory connections. The population of CCs was divided into a larger subpopulation that received olfactory inputs from the MCs and a smaller subpopulation that was driven by non-olfactory, contextual input. This allowed this area to learn the association of specific odors with specific contexts. To capture the experimentally observed excitation of GCs by non-olfactory context information associated with specific learned odors [26], we implemented top-down projections from these CCs to the bulbar GCs. At this point it is not known whether these projections arise directly from the neurons involved in the associative pattern completion or whether additional cortical neuron populations are involved. Thus, the CCs were not meant to model a specific cortical neuron population; instead, they were intended as an effective neuronal population that mimics the observed pattern completion and the non-olfactory excitation of GCs. For simplicity, we call these CCs ‘cortical cells’.

**Fig 1:**
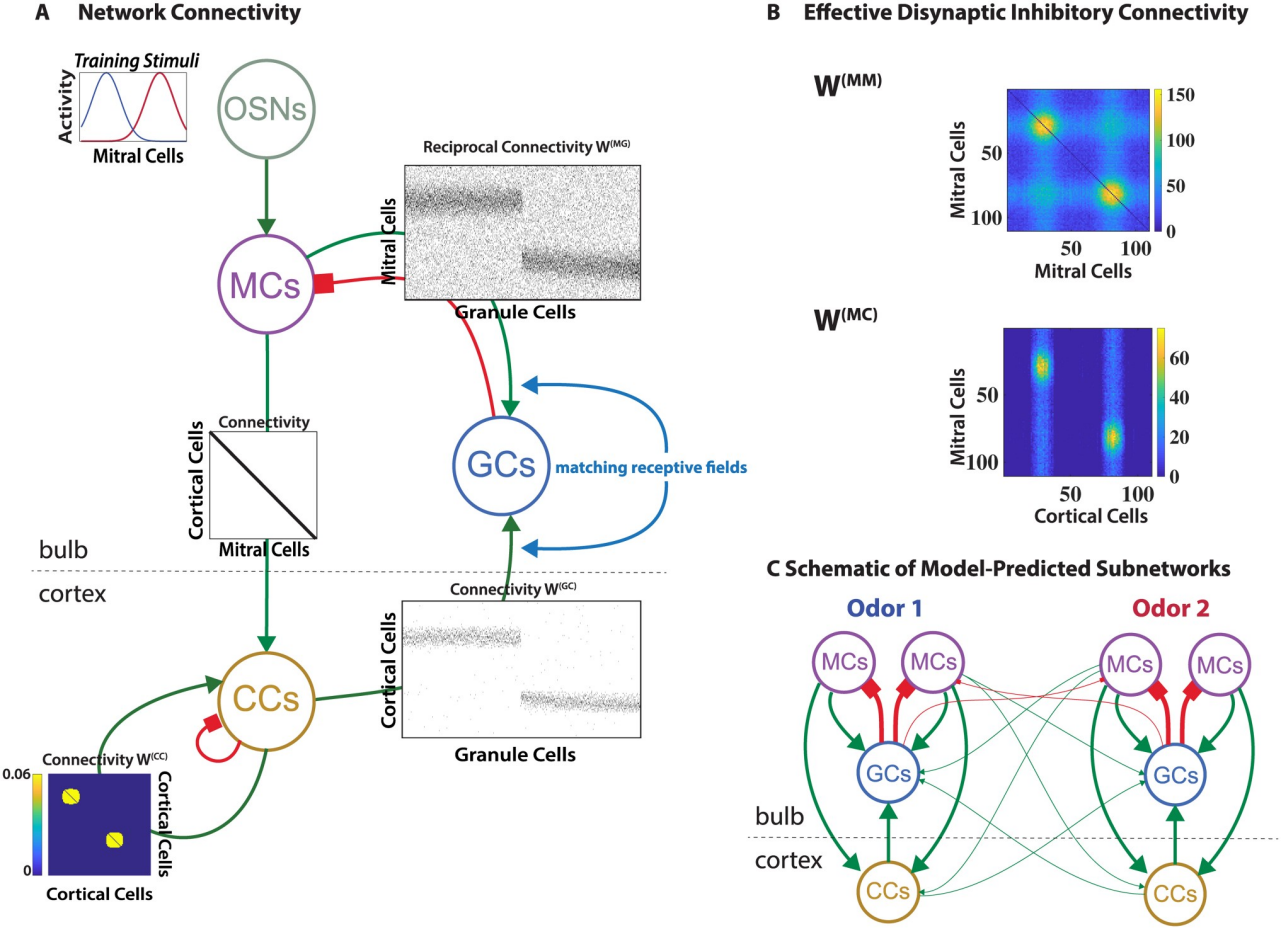
Computational model. (A) Network structure emerging after learning 2 training stimuli. The modeled neuronal populations are indicated by circles marked OSNs (sensory neurons), MCs, GCs, and CCs. Excitatory connections in green with arrows, inhibitory ones in red with squares. Connectivity matrices indicate the connectivities between the individual neurons of the populations (black = connection, white = no connection; cf. Fig 2). For the intra-cortical connectivity *W*^(*CC*)^ the colors indicate the synaptic strength of the connections. (B) The GCs mediated disynaptic mutual inhibition of MCs with effective connectivity matrix *W*^(*MM*)^ and disynaptic inhibition of MCs by CCs with effective connectivity matrix *W*^(*MC*)^. The color indicates the number of GCs contributing to the respective connections. (C) Idealized schematic of the network structure emerging after learning two odors: CCs disynaptically inhibit predominantly those MCs that respond to the same odor as the CCs. Line thickness indicates the number of connections.

**Fig 2:**
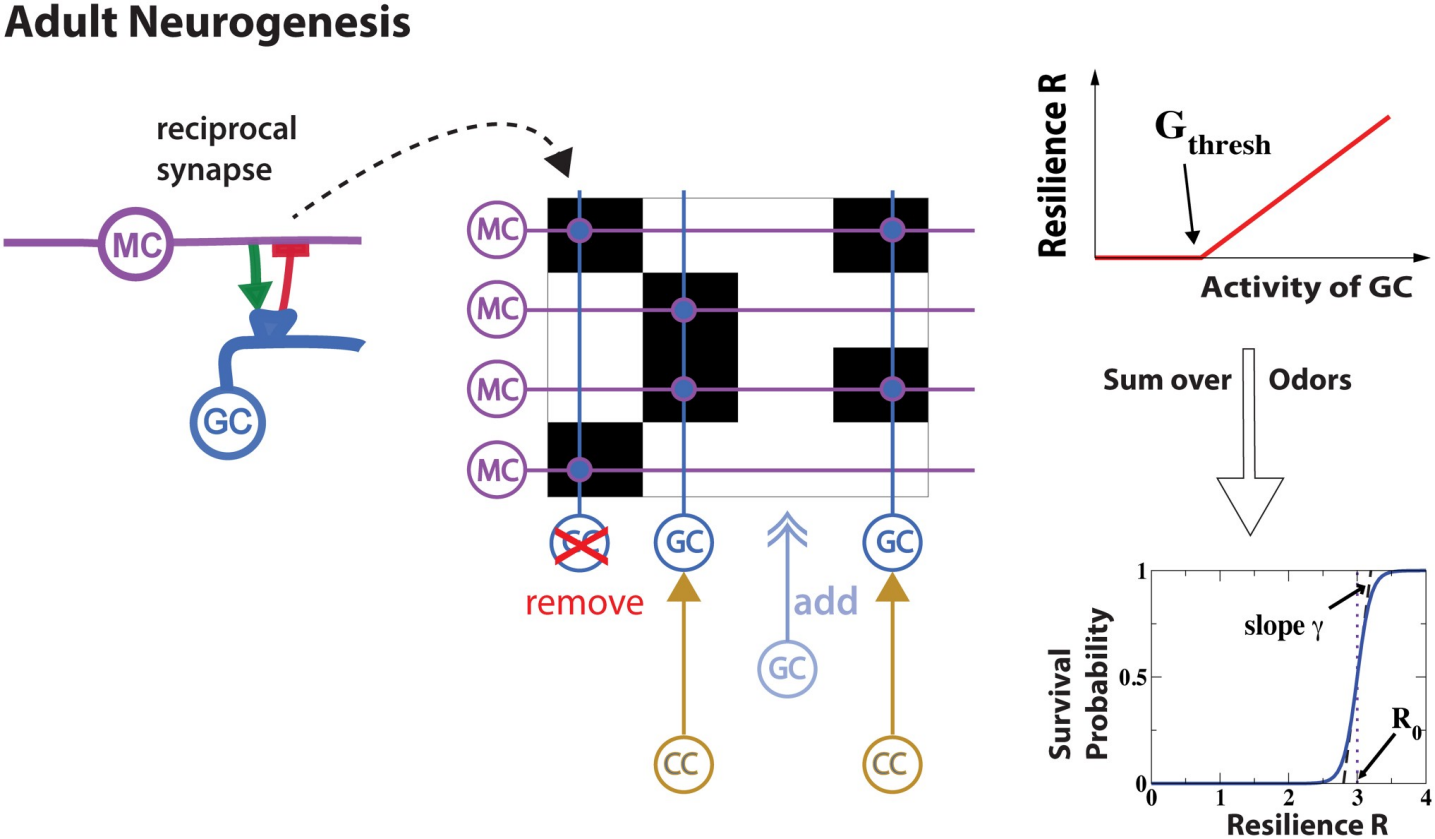
Model of adult neurogenesis. In each time step adult neurogenesis added GCs, which made random reciprocal synapses with MCs: the synapses excited the GCs and inhibited the MCs that they connected (left panel). In addition, the GCs received excitatory projections from random CCs. The synapses between the individual MCs and GCs are indicated by black rectangles in the connectivity matrix (middle panel). GCs were removed reflecting a survival probability that depended on GC-activity via a resilience that was summed over the training stimuli (right panel).

In Fig 1A each neuron population is indicated by a circle and the connections between the individual neurons of the various populations are shown in terms of connectivity matrices (black = connection, white = no connection between the respective neurons as illustrated in Fig 2 below). The MCs received excitatory input from the sensory neurons (OSNs) and formed excitatory projections to the CCs as well as reciprocal synapses with the inhibitory GCs. This reciprocal nature of the MC-GC synapses is essential for the function of the system. To aid visualization of the results, in most computations each CC received input from only one MC, resulting in a diagonal connectivity matrix. In the Supporting Information S6 Fig we also show results for the more realistic case of an expansion from the bulb into the cortex, which resulted in a sparse cortical odor representation. The CCs formed all-to-all recurrent excitatory connections among themselves, which were endowed with Hebbian plasticity to give this network autoassociative properties. In addition, they inhibited each other via an unmodeled interneuron population. The CCs’ top-down projections formed excitatory synapses onto the GCs.

All neurons were described using nonlinear firing rate models. For simplicity, the MCs and GCs had threshold-linear firing-rate functions. The MCs exhibited some spontaneous firing in the absence of odor input, while the GCs responded only when their combined input from MCs and CCs surpassed a positive threshold. The saturation of the CC-response needed for their associative property was implemented with a sigmoidal nonlinearity.

The key aspect, network restructuring by adult neurogenesis, was implemented by persistently adding and removing GCs, as sketched in Fig 2. Newly added GCs formed randomly chosen connections with a subset of the MCs and of the CCs. Note that Fig 2 depicts only the reciprocal connections between GCs and MCs, overlaid onto the corresponding connectivity matrix. GCs were removed with a probability that decreased sigmoidally with their ‘resilience’. The resilience of a GC was taken to be the sum of its thresholded activities, which were driven by MCs and CCs in response to a set of training stimuli. This activity-enhanced survival of GCs was motivated by a host of experimental observations. Odor deprivation up-regulates GC cell death [21, 36], while odor enrichment increases the number of GCs [37]. More direct, physiological evidence is provided by genetic experiments in which the excitability of GCs was enhanced and reduced by modifying *Na*- and *K*-channels, respectively [38]. This enhanced and reduced the survival of GCs, correspondingly. The enhancement of activity could even rescue GCs from cell death induced by sensory deprivation [38].

The network structure arising in this model for a range of suitable parameter values is illustrated in Fig 1A and 1B for a simple set of two training stimuli that activated partially overlapping sets of MCs. We mostly used such simplified odor-evoked patterns rather than natural stimuli [19, 39], because they facilitated the visualization and understanding of the emerging network connectivities. Results using natural stimuli based on odor maps obtained by Johnson and Leon (cf. [40]) are presented in Supporting Information S3 Fig. Since all connections were learned based on activities, the spatial arrangement of the various neurons played no role in the model. Exposing the network to these odors lead to a strengthening of the recurrent connections among the CCs that were co-activated by the odors, implementing an associative memory of these odors. Due to the non-zero threshold for GC activation, for a GC to survive it needed to be co-activated by multiple MCs and CCs at least for some of the training stimuli. Thus, the surviving GCs were connected predominantly to MCs and CCs that had similar receptive fields.

**Fig 3:**
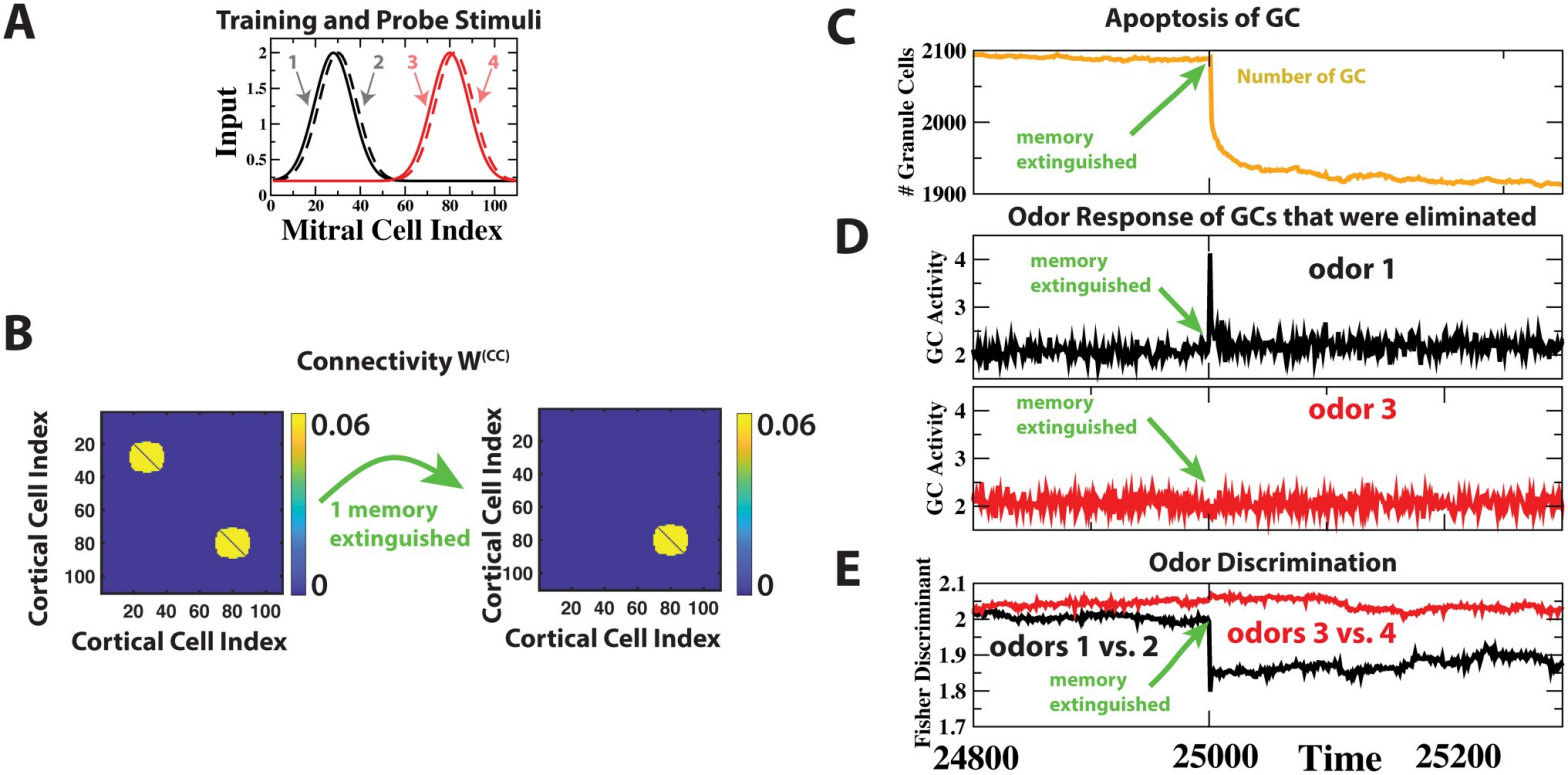
Extinction of cortical odor memory eliminates GCs. (A) Training and probe odors. (B) Extinguishing the cortical memory of odors 1 and 2 was implemented by removing the associative connections between the respective CCs. (C) Extinction induced the apoptosis of many GCs. (D) In each time step we measured the average of the odor response across the GCs that were to be removed in that time step. On average, the GCs that were removed as a result of the memory extinction had strongly responded to odor 1 before the extinction, but not to odor 3. (E) The extinction-induced reduction in inhibition compromised the discrimination between the ‘forgotten’ odors 1 and 2, but not between odors 3 and 4.

To wit, in Fig 1A one population of GCs was mostly driven by MCs and CCs responding to the left training stimulus, while the other population responded to the one on the right. Due to the reciprocal nature of the MC-GC synapses [41] this induced not only mutual disynaptic inhibition of MCs that are co-activated by some of the training stimuli [19, 39] but also inhibition of MCs by cortical CCs that shared that receptive field. The effective connectivity matrices for the disynaptic inhibition among MCs and of MCs by CCs are shown in Fig 1B. Due to the persistent addition and removal of GCs these connectivities fluctuated in time leading to trial-to-trial variability, as discussed in Supporting Information S4 Fig. This trial-to-trial variability is also apparent in the temporal fluctuations of the number of GCs and the odor response and odor discriminability shown in Fig 3C, 3D and 3E below.

Thus, the emerging network structure is characterized by subnetworks or network modules, each of which is associated with a learned odor and provides a bidirectional projection between the bulbar and cortical representation of that odor. This is idealized in Fig 1C, where the circles again represent neuronal populations and the thickness of the lines indicates the number of neurons involved in that type of connection. Through this cortico-bulbar feedback structure cortical cells have inhibitory control specifically over those MCs that provide their dominant sensory input. Of course, depending on the similarity of the odors in the training set their associated subnetworks are more or less overlapping or intertwined.

The simulation experiments presented in the body of this paper are for one set of model parameters. Extensive further analysis has shown, however, that our results, particularly for the network structure, are robust with respect to parameter changes (cf. Supporting Information S5, S7, S8, S9 Figs).

What ramifications does this odor-specific network structure have for stimulus processing? In the following we use simulation experiments to address this question in a number of behaviorally relevant settings.

### Perceptual learning of odor discrimination

Behavioral experiments on spontaneous odor discrimination using a habituation protocol have shown that exposure to an odor related to the odors used in the discrimination task can induce a perceptual learning of that task; however, this learning was compromised when adult neurogenesis was suppressed [24]. A parsimonious interpretation of this finding is that the restructuring of the bulbar network by adult neurogenesis enhances differences in the bulbar representations of the similar odors rendering them more discriminable. To assess the impact of the network structure on odor discriminability we envisioned a read-out of the bulbar output that consists of the sum of the suitably weighted outputs of all MCs. Discriminability can then be characterized by Fisher’s linear discriminant F given by the square of the difference between the trial-averaged read-outs corresponding to the two odors divided by the trial-to-trial variability of the read-outs. Our firing-rate framework did not include any trial-to-trial variability. We therefore took as a proxy for it the firing rate, which would be proportional to the variability if the rates arose from Poisson-like spike trains. We considered here the optimal value $F_{opt}$ that is obtained if the weights of the outputs to the read-out are chosen to maximize F for the stimuli in question. Such optimal weights could be the result of the animal learning the task. For similar odors $F_{opt}$ typically increased in our model as the network structure evolved in response to these odors, typically in parallel with a reduction in the Pearson correlation of the MC activity patterns, capturing the observed perceptive learning [24] (cf. our previous results for a purely bulbar model [39]).

We assessed network performance based on the MC activity patterns for two reasons. As mentioned above, through the MC projections the information flows from the bulb to a variety of other brain areas [27–29], each of which making use of that information for different purposes. The MC activity patterns constitute therefore the central basis for multiple types of odor processing, and enhancements of the bulbar odor representations will affect all of them. Moreover, the CCs included in our model represented only a single aspect of such processing: associating odors with contexts [26] and associative pattern completion [30–32]. Thus, the CC-activity had a very strong contextual *excitatory* component, which was independent of the odor stimuli. The impact of contexts on CCs was therefore opposite to that on MCs and reduced the discriminability of these CC-patterns (cf. Supporting Information S10 Fig). These CCs were therefore not suitable as a read-out that aims to discriminate very similar odors. We envision that the modeled CCs effectively represent only a subpopulation of cortical cells, while other cortical cells, which were not included in our model, are engaged in tasks like fine discrimination of odors. This interpretation is in part motivated by the fact that the balance between feedforward sensory input and recurrent associative input varies along the anterior-posterior axis of piriform cortex [42] and between its types of principal cells [43, 44] as well as by recent observations indicating that different populations of cells in anterior piriform cortex encode odors differently [45].

### Odor-specific apoptosis after extinction of odor memory

The survival of the model GCs depended on the sensory input they received from the MCs as well as on the top-down input from the CCs. The top-down input was enhanced if the presented odor had previously been ‘memorized’, i.e. if the corresponding recurrent excitatory connections among the CCs had been strengthened. What happens if the cortical memory of one of the learned odors is erased, i.e. if the recurrent connections of the CCs representing that odor are removed? In our simulations removal of the memory of odor pair 1 (Fig 3B) significantly enhanced cell death and quickly reduced the total number of GCs (Fig 3C). The GCs that were removed during this phase were predominantly GCs that had previously responded to the odors in that pair and whose top-down inputs had been enhanced by the cortical memory of that odor pair (Fig 3D and Supporting Information S1 Fig). In parallel, the network’s ability to discriminate between the odors in that pair was substantially reduced (Fig 3E). This degradation of the performance did not occur if the removal of GCs was blocked in the simulation.

These results capture essential features of experiments in mice in which the extinction of an odor memory enhanced the apoptosis of GCs, particularly of those GCs that had been responsive to that odor. However, fewer of the GCs died and the mice did not forget the task when apoptosis was blocked during the extinction of the odor memory [25].

### Specific GC activation in the absence of odor stimulation

Experimentally it has been found that specific GC activity patterns could be evoked even in the absence of odors, if the animal was placed in an environment that previously had been associated with an odor [26]. In fact, the GC activation pattern that was induced by this environment had substantial overlap with the pattern evoked by the associated odor. This was not the case for a different environment. A natural interpretation of these observations is that the odorless GC activation patterns were driven by top-down projections onto the GCs from higher brain areas that have access to non-olfactory, e.g. visual, information [26]. To capture this aspect we extended our cortical model to include CCs that did not receive direct input from the olfactory bulb but were driven by non-olfactory, contextual information. This could, for instance, represent information from other sensory modalities, information about the task the animal is to perform, or an expectation by the animal. We introduced excitatory associational connections with Hebbian plasticity between these cells and the CCs that received MC input and extended the global inhibition to those cells.

**Fig 4:**
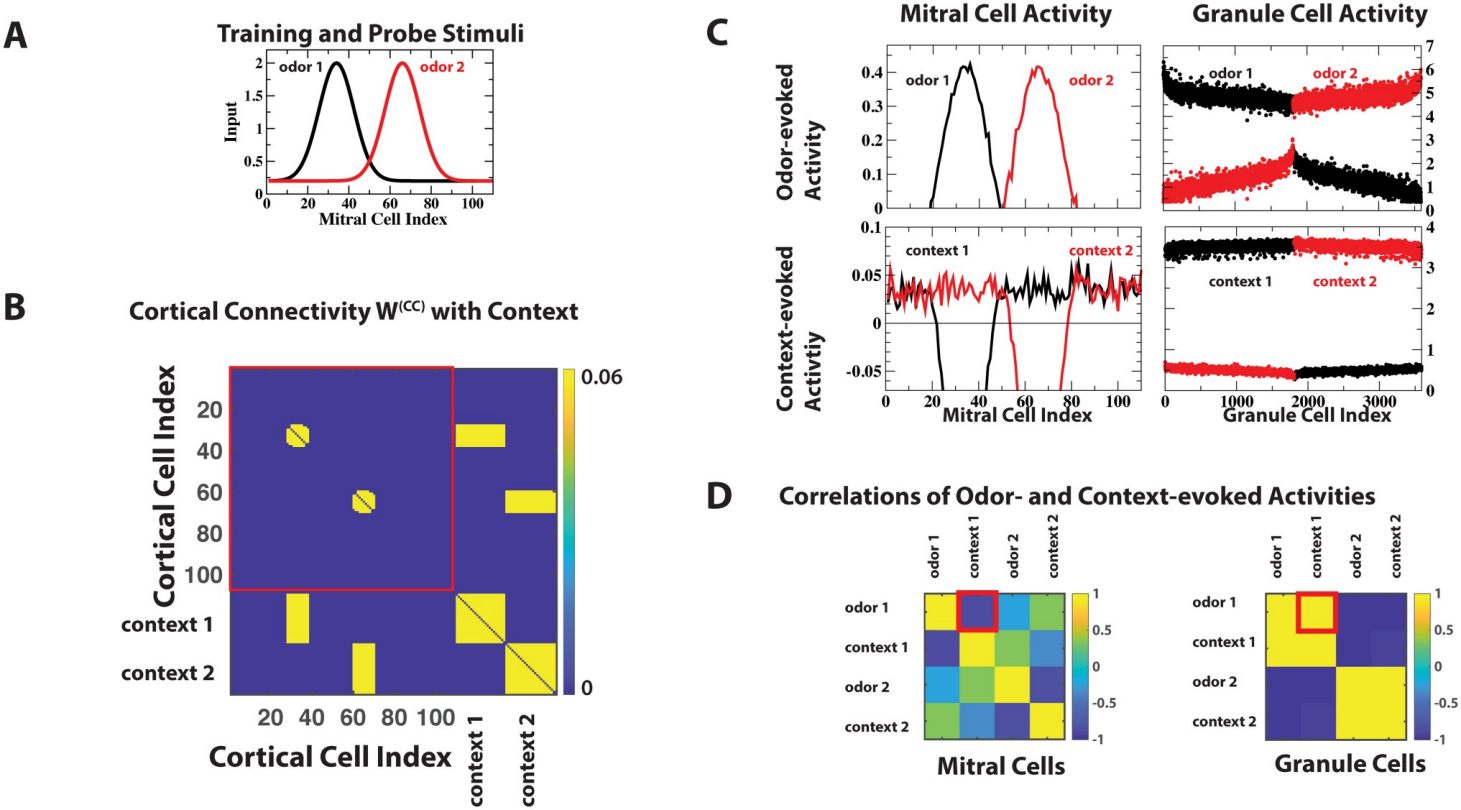
Context induces specific GC activity. (A,B) The 2 training stimuli were associated with contexts 1 and 2, respectively. In (B) CCs with index up to 110 received odor information from MCs. CCs with index above 110 received contextual information. (C) Upper panel: odor-evoked MC- and GC-activities (odor 1: black lines, odor 2: red lines). Lower panel: context-evoked MC- and GC-activities (context 1: black lines, context 2: red lines). (D) Context- and odor-evoked MC activities were anti-correlated, while context- and odor-evoked GC activities were highly correlated (red boxes in (D)).

Presenting two odors to the network, each in the presence of a different context, established associational connections in the cortical network between the CCs that received odor input (cell indices 1 to 110) and the CCs that received the corresponding contextual input (marked ‘context 1’ and ‘context 2’ in Fig 4B) This enabled the contextual input to excite GCs via top-down projections (Supporting Information S2 Fig) even in the absence of any odor stimulation. Due to the specificity of the cortico-bulbar network the resulting odorless GC activity patterns were highly correlated with the patterns induced by the associated odor, but not with those of the other odor (Fig 4C and 4D), recapitulating the experimental observation. In contrast, the context-evoked, odorless MC patterns were anti-correlated with the odor-evoked patterns, akin to representing a novel ‘negative’ odor, which may evoke a very different percept than the odor itself. This may account for the enhanced response of the animals observed in the familiar, but odorless context, but not in the unfamiliar context [26].

What functionality is enabled by the learned network structure, which allows CCs to inhibit specific MC? To assess this question we considered two scenarios: i) the detection and discrimination of odors in a cluttered environment and ii) rapid switching between different odor tasks.

### Context enhances detection and discrimination in cluttered odor environments

**Fig 5:**
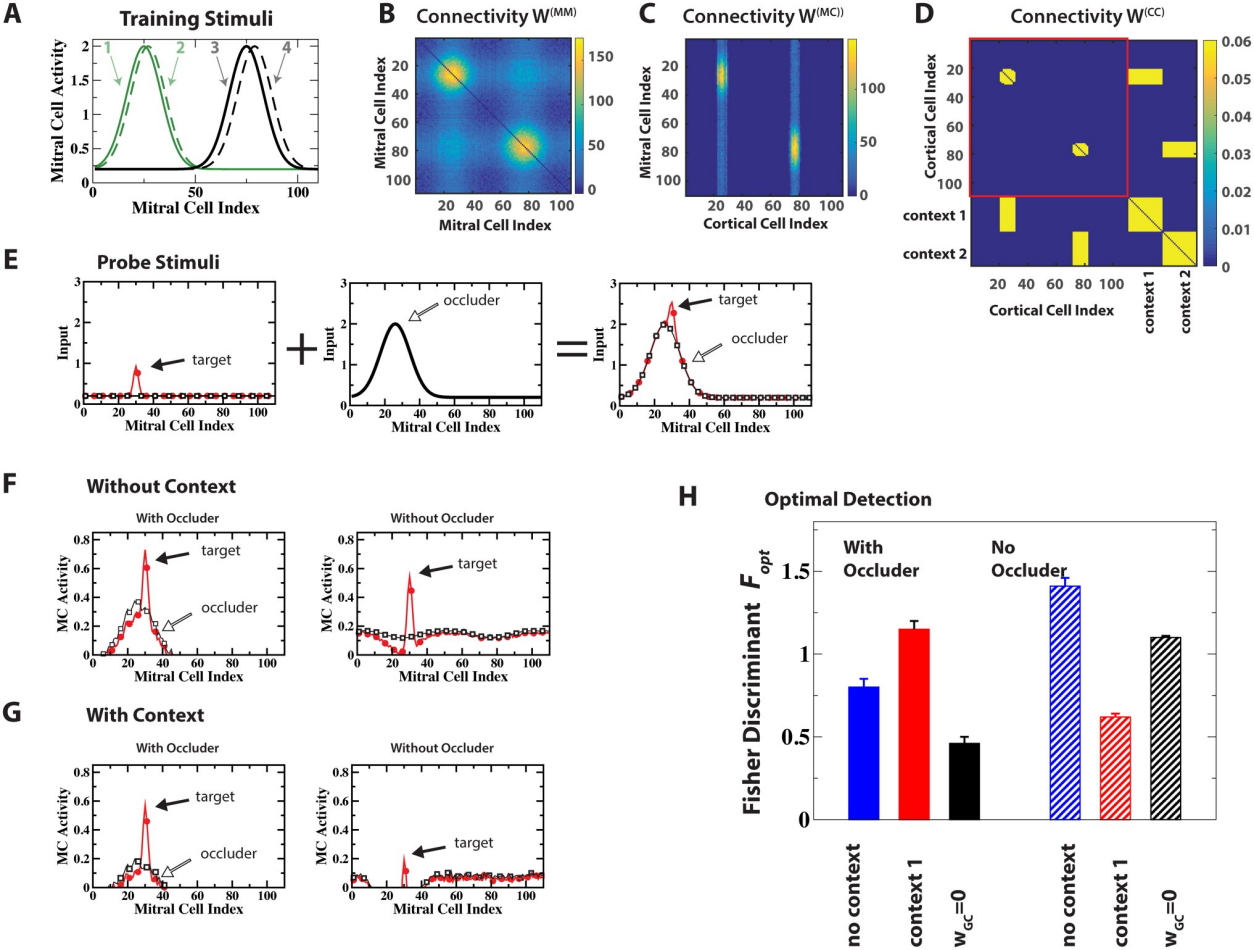
Context-enhanced odor processing in cluttered environments: Occluding stimulus. (A,D) Training stimuli 1,2 and 3,4 were associated with contexts 1 and 2, respectively. (B,C) Learned disynaptic inhibitory connections among MCs (*W*^(*MM*)^) and from CCs to MCs (*W*^(*MC*)^). The color indicates the number of GC mediating the respective connections. (E) Probe stimuli consisted of a weak target odor (red line) with or without stimulus 1 as a strong occluding odor (black line). (F,G) MC activities resulting from the probe stimuli with and without context 1. Red: target with occluder, black: occluder alone. Context 1 reduced the response to the occluder, enhancing the detectability of the target. (H) Context 1 increased the Fisher discriminant $F_{opt}$ when the occluding odor was present, but reduced $F_{opt}$ when it was absent. With top-down input blocked (*w*_*GC*_ = 0) the occluder is suppressed even less than without any context, deteriorating the performance further. The error bars indicate the standard deviation in $F_{opt}$ resulting from fluctuations in the network connectivity.

We considered cluttered environments in which the presence of additional odors may or may not occlude (mask) the odors of interest, an olfactory analog of the ‘cocktail party’ problem [46]. As an illustrative example we considered the detection of a weak target odor (Fig 5). In the presence of a strong odor that activated a large number of MCs and occluded the target odor this required the discrimination between the occluder alone and the occluder with the target (Fig 5E). This was difficult because the MCs carrying the information about the target were also driven by the occluder, rendering the relative difference between the overall pattern with target (red, solid symbols) and that without target (black, open symbols) small. If the activation by an occluding odor could be reduced without suppressing the contribution from the target odor, detection of the target should be significantly enhanced. Indeed, in our model such an odor-specific inhibition was possible if the occluding odor was familiar, i.e. if it had been one of the training stimuli.

In these simulation experiments we associated the occluding odor during the training with context 1 (Fig 5D). The odor-specific inhibition then had two contributions. The intra-bulbar component did not require cortical activation and suppressed the familiar occluder on its own (Fig 5F left panel). In the presence of the context the detectability of the target was even further enhanced by the cortical excitation of the GCs (Fig 5G left panel), increasing the Fisher discriminant $F_{opt}$(Fig 5H). However, the same context was detrimental for the detection of the target odor if the occluding odor was not present: the strong context-enhanced inhibition almost eliminated the response to the target odor (Fig 5F and 5G right panels). The flexibility in the control afforded by top-down inputs therefore substantially enhanced performance.

The activity of the CCs and their inputs to the bulb play dual roles: they shape the cortical-bulbar connectivity during the network development and they provide—via that connectivity—input to the bulb during the task performance. To illustrate the impact of the established connections from the CCs to the GCs, we include in Fig 5H the outcome when the top-down input was blocked (*w*_*GC*_ = 0) after the network had been established. Even in the absence of the context signal the CCs representing the familiar, occluding odor were excited, albeit less strongly. Blocking their input to the bulb therefore disinhibited the MCs representing the occluding odor and reduced the Fisher discriminant. Blocking the top-down input in the absence of the occluder avoided the excessive suppression of the target odor by the context, leading to a larger Fisher discriminant. However, the discrimination turned out not as good as in the presence of top-down input but without the context signal, since that top-down input also reduced the background activity of the MCs.

**Fig 6:**
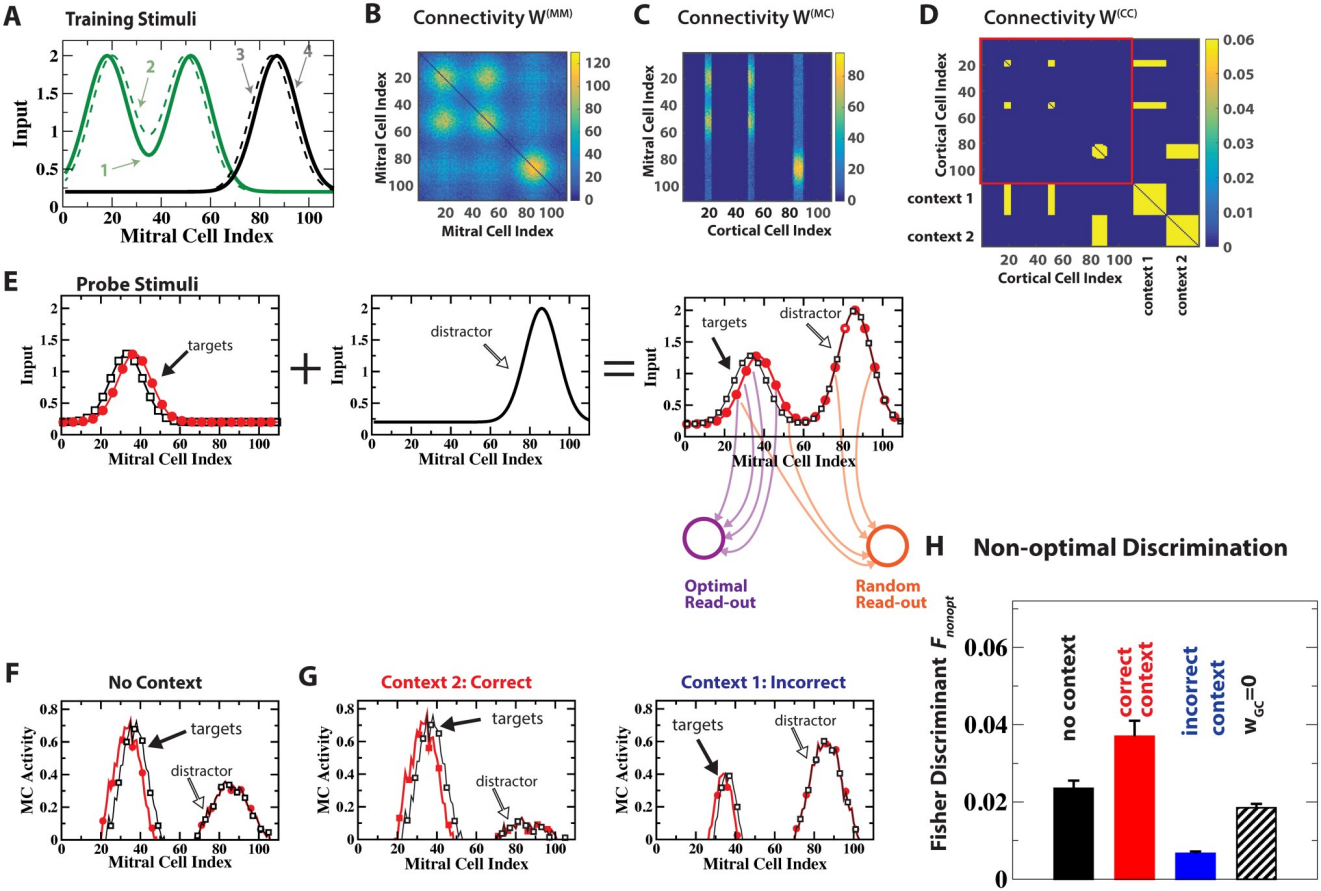
Context-enhanced odor processing in cluttered environments: Distracting stimulus. (A,D) Training stimuli 1,2 and 3,4 were associated with contexts 1 and 2, respectively. (B,C) learned disynaptic inhibitory connections among MCs (*W*^(*MM*)^) and from CCs to MCs (*W*^(*MC*)^). Colors indicate the number of GCs mediating that inhibition. (E) The probe stimuli (right panel) consisted of a pair of similar, weaker target odors (open black and solid red symbols, left panel) on top of training stimulus 3 (black solid line without symbols, middle panel) as a strong distractor. Sketch of connections indicate an optimal read-out that focuses on the target odors and a non-optimal, random read-out. (F,G) MC activities in response to the probe stimuli with and without context. (H) The correct context suppressed the distractor and enhanced the Fisher discriminant $F_{nonopt}$ of the random read-out. In contrast, the incorrect context reduced $F_{nonopt}$. With top-down input blocked (*w*_*GC*_ = 0) $F_{nonopt}$ is slightly reduced compared to the context-less case. The error bars quantify the fluctuations in the network connectivities.

If the odors of interest are not occluded by any other odors in the environment, read-out cells can, in principle, adapt the weights of their input synapses so as to focus only on the relevant odors and ‘ignore’ the cluttered environment. However, this weight optimization is often not possible, since the animal may not know yet which odors are to be discriminated. This is, for instance, likely the case in the early phase of learning a new discrimination task [35]. During this phase it is reasonable to envision that animals rely on a large number of read-out cells each of which receives inputs from different combinations of MCs and is therefore sensitive to different aspects of the MC activity patterns. The activity of many of these read-out cells will be dominated by the uninformative components of the odor environment (‘distractor’), making it difficult to discriminate between two weak target odors, even if they are not occluded by the environment (Fig 6E, right panel illustrating optimal and random read-out). To analyze such a situation we employed a non-optimal Fisher discriminant $F_{nonopt}$ based on a large number of random read-outs of the MCs (cf. [10] in Methods).

We considered the discrimination between two similar, novel target odors in the presence of a strong, familiar odor that did not occlude the targets but served as a distractor (Fig 6E). It was associated with context 2. In addition, the network was familiarized with odors 1 and 2, which were associated with context 1 and partially overlapped with the novel target odors (Fig 6A–6D). Even in the absence of any contextual signal the learned intra-bulbar connectivity was able to suppress to some extent the distracting, familiar odor relative to the novel odors (Fig 6F). In the presence of context 2, which was associated with the distractor (‘correct’ context), this suppression was substantially enhanced through the cortical feedback driven by that context, leading to much better discriminability of the two novel odors (Fig 6G and 6H).

As in the case of discrimination via an optimal read-out (Fig 5) it was not beneficial to have indiscriminate strong cortical feedback for all familiar odors, even if these odors were not present. For instance, the feedback driven by context 1 (‘incorrect’ context) was detrimental to the discrimination of the target odors if neither of the familiar odors 1 and 2, which were associated with context 1, were part of the odor scene. While the target odors were not occluded by these familiar odors, they had significant overlap with them. Therefore the connectivity that was learned through the training included a sizable number of inhibitory projections—via the GCs—from the CCs representing context 1 to the MCs representing the target odors. This lead to a strong suppression of the MC response to the target odors, resulting in poor discriminability (Fig 6G and 6H). If the top-down input was blocked (*w*_*GC*_ = 0), neither the beneficial nor the detrimental impact of the two contexts arose.

Thus, the ability of top-down inputs to induce specific inhibition in a *flexible* manner substantially enhanced olfactory processing.

### Top-down input enables task switching

The neurogenic evolution of the structure of the cortico-bulbar network occurs on a time scale of days, particularly since the apical reciprocal MC-GC synapses form only days after the proximal synapses of the top-down projections [47]. Synaptic plasticity based on changes in the synaptic weights can act on much shorter time scales, allowing cortical associational connections to adapt faster to changes in the tasks that the animal needs to perform. This could modify the top-down signals to the bulb, altering bulbar processing.

**Fig 7:**
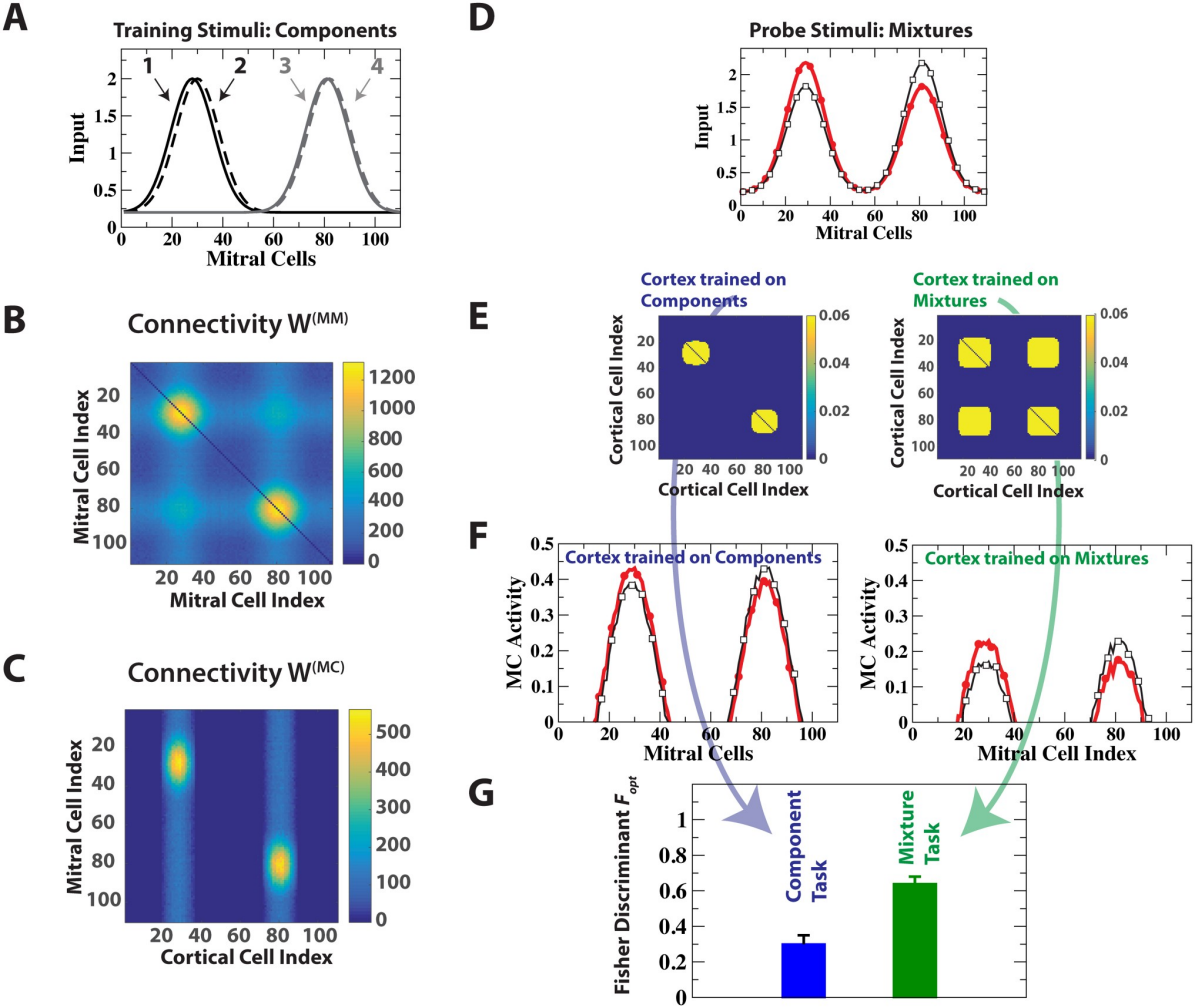
Cortical task switching enhances discrimination. (A) The training stimuli consisted of 2 pairs of dissimilar stimuli. (B,C) Resulting bulbar and cortico-bulbar connectivity. (D) The probe stimuli consisted of two mixtures of the training stimuli. (E) Cortical connectivities after cortical training on the individual components (left) and on the mixtures (right). (F) MC activities with cortex trained on the components (left) and on the mixtures (right), respectively. After training on the mixtures the inhibition by the top-down input reduces the MC amplitudes without reducing the difference between the two mixtures, enhancing their discriminability. (G) Optimal Fisher discriminant $F_{opt}$ with cortex trained on the components (blue) and on the mixture (green). The error bars reflect the ongoing network fluctuations.

As an example we considered a situation in which the network was trained on two pairs of odors, *O*_1,2_ and *O*_3,4_. The two odors in each pair were very similar, but the pairs were dissimilar from each other (Fig 7A). As expected, the training increased the discriminability of the odors within a pair. However, for the discrimination of the mixture *M*_1_ = 0.55*O*_1_ + 0.45*O*_3_ from the mixture *M*_2_ = 0.45*O*_1_ + 0.55*O*_3_, obtained by combining odors from the two pairs, the training on the individual components *O*_1,2_ and *O*_3,4_ was detrimental. While it established mutual inhibition of MCs that were activated by the *same* mixture component (*O*_1_ *or* *O*_3_), it provided only little mutual inhibition between MCs that were activated by different mixture components: the number of connections between MCs representing odor *O*_1_ (MCs with index near 30 in Fig 7B) and MCs representing odor *O*_3_ (MC index near 80) was small. However, these MCs are activated simultaneously in the mixtures and mutual inhibition of these MCs is needed to enhance the discrimination between the mixtures [19, 39, 48, 49]. As a result the inhibition significantly reduced the relative difference between the two mixtures and with it their Fisher discriminant (Fig 7F, left panel, and Fig 7G). Inhibition between the MCs representing *O*_1_ and those representing *O*_3_ can be effected by establishing associational excitatory connections between the CCs that disynaptically inhibit these two groups. To do so we exploited the Hebbian plasticity of the cortical synapses and trained the cortical network briefly on the mixture 0.5*O*_1_ + 0.5*O*_3_ (Fig 7E, right panel). This enhanced the discriminability of the mixtures substantially (Fig 7F, right panel, and Fig 7G). The role of the top-down inputs before and after learning are qualitatively different. Before learning, the intra-bulbar as well as the cortically driven inhibition of mixture component *O*_1_ increases with *O*_1_-activation, which reduces the difference in the *O*_1_-activity in the two mixtures, and analogously for component *O*_3_. This reduces the discriminability of the mixtures. After learning, the cortical contribution to the inhibition of each component is driven by the combined activity of both components and is therefore identical for both mixtures. This preserves their difference. In S11 Fig (Supporting Information) we show that this improvement is not due to the overall reduction in MC-pattern amplitude.

Thus, cortical projections can exploit the learned, odor-specific subnetwork structure (Fig 1C) to switch between different cortico-bulbar processing modes, adapting to the odor objects at hand.

### Dependence of connectivity and function on the strength of the top-down input

Since the top-down input is at the core of the bidirectional mapping between the bulbar and cortical odor representations, we considered the influence of its strength *w*_*GC*_ on the connectivity and function of the bulbar-cortical network in some detail. Here we focus on the context-enhanced processing of a stimulus in the presence of a distractor. The results for an occluded stimulus are presented in S9 Fig in Supporting Information.

**Fig 8:**
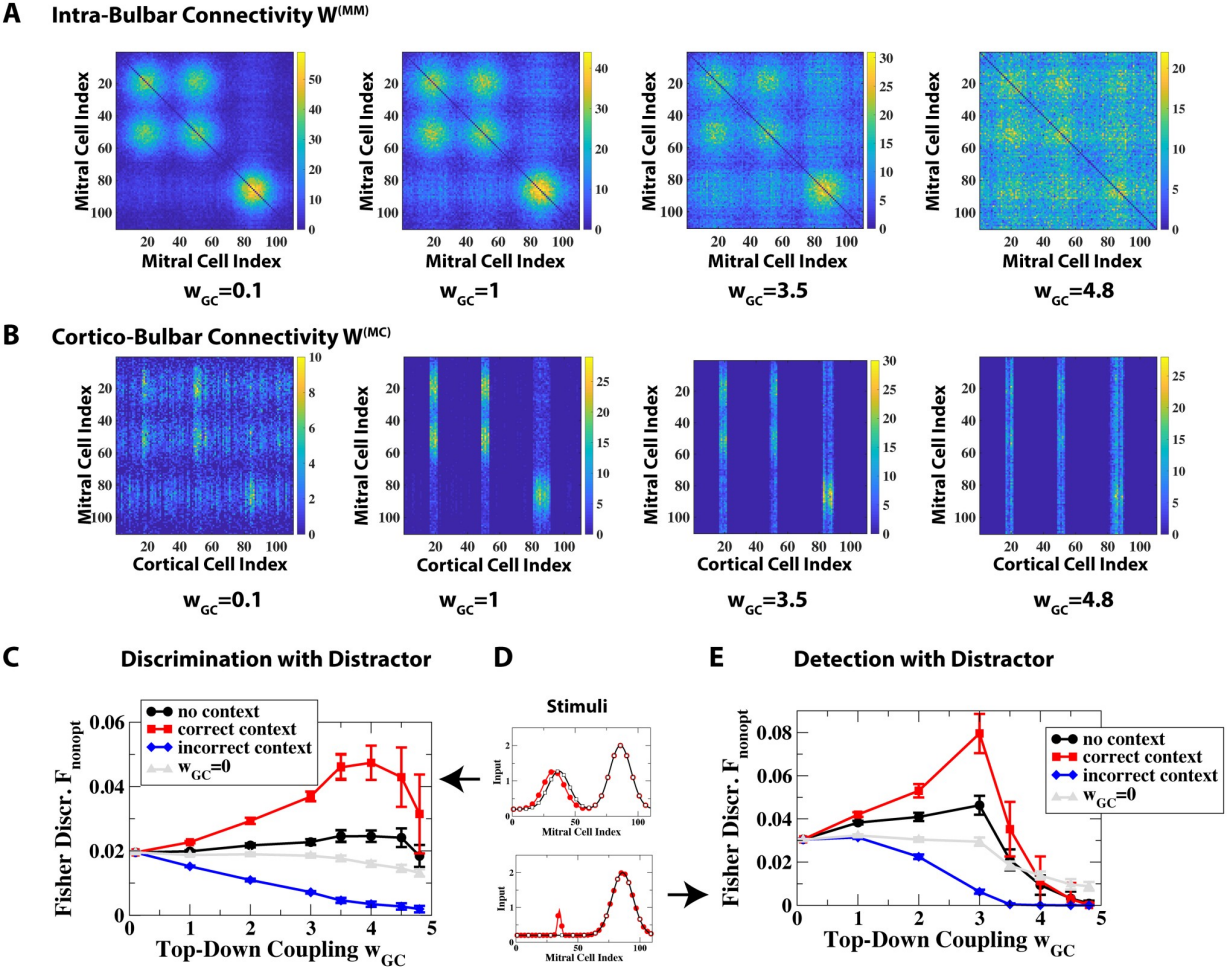
Role of top-down input in the discrimination and detection of stimuli with distractor. The system was trained with the stimuli of Fig 6A. (A) Intra-bulbar disynaptic inhibition among MCs (*W*^(*MM*)^) became less selective with increasing top-down weight *w*_*GC*_. (B) The disynaptic inhibition of MCs by CCs (*W*^(*MC*)^) was selective for an intermediate range of *w*_*GC*_, but not for small or large values of *w*_*GC*_. (C) Fisher discriminant $F_{opt}$ for the non-optimal discrimination of two similar stimuli in the presence of a distractor (cf. Fig 6 and upper panel of (D)) as a function of *w*_*GC*_. The correct context enhanced the discrimination only when the connectivities *W*^(*MM*)^ and *W*^(*MC*)^ were selective. Even with the top-down input blocked by setting *w*_*GC*_ = 0 during the probing (but not during the network development) the discriminability of the odors was reduced when *w*_*GC*_ was increased during the learning (grey line), reflecting the decrease in selectivity of *W*^(*MM*)^. (D) Stimuli used for probing in (C,E). (E) Fisher discriminant $F_{opt}$ for the non-optimal detection of a weak stimulus in the presence of a distractor. Grey line: *w*_*GC*_ = 0 during probing but not during training of the network.

The top-down inputs are instrumental during the network development as well as during the processing of stimuli. The survival of GCs depends on their overall activity, which results from bulbar and from cortical inputs. Therefore, increasing the strength *w*_*GC*_ of the top-down projections shifts the balance from the bulbar inputs determining the survival and with it the network connectivity to the cortical inputs dominating the development. This is demonstrated in Fig 8 for the cluttered environment investigated in Fig 6. For very small *w*_*GC*_ training with the stimuli shown in Fig 6A resulted in a highly selective intra-bulbar connectivity in which the mutual inhibition was essentially restricted to MCs that were co-active for one of the training stimuli. The MCs received, however, disynaptic top-down inhibition that only slightly reflected the receptive fields of the MCs and the CCs, and most CCs induced inhibition on most MCs that responded to *any* of the training stimuli (Fig 8B). In the opposite limit of large *w*_*GC*_ only few CCs had projections to the bulb, but the inhibition they induced was only weakly targeted to specific MCs. Moreover, the intra-bulbar connectivity was essentially homogeneous. As a result, the inhibition induced by the top-down projections did not allow different CCs to inhibit specific different sets of MCs. Thus, to obtain a subnetwork structure of the type sketched in Fig 1C, *w*_*GC*_ had to be in an intermediate range in which the bulbar and cortical inputs to the GCs were of similar magnitude (1 ≲ *w*_*GC*_ ≲ 3.5 in Fig 8).

We probed the performance of the resulting networks with two tasks. In both, the stimuli involved a distractor that is very different from the target odors (Fig 8D) and we considered the non-optimal read-out discussed in Fig 6. For the context associated with the distractor to enhance the processing, the top-down input has to suppress the MC-activity driven by the distractor but not that due to the target odors. This was the case for intermediate values of *w*_*GC*_ and resulted for both types of target odors in an increase in $F_{nonopt}$ in the presence of the correct context, but in a decrease for the incorrect context (Fig 8C and 8E). For larger *w*_*GC*_, however, the performance deteriorated, both with the correct context and without any context. This was caused by the decrease in the specificity of the connectivity, which is quantified in S7 Fig. As a result even the correct context suppressed the activity of the MCs responding to the target odors, reducing $F_{nonopt}$. This is shown explicitly in S8 Fig in Supporting Information. A similar dependence on *w*_*GC*_ was found for the optimal detection of an occluded target (S9 Fig in Supporting Information, cf. Fig 5).

### Experimental predictions

The key anatomical feature of the model network resulting from learning is its connectivity. Specifically, the projections that the GCs receive from the MCs and from the CCs are predicted to be matched: a given GC receives cortical inputs predominantly from those CCs that respond to the same odors as the MCs projecting to that GC. This matching of the GCs’ receptive fields can be tested experimentally. One possibility is to express—after suitable training—ChR2 conditionally (e.g., via c-Fos [50]) in those principal cells of piriform cortex that are activated by the training odor, combined with expression of a calcium-indicator in the GCs. Note that the training needs to activate the neurogenic plasticity of the bulb [24], which is not the case if the odor exposure is only passive [51]. The model predicts that optical stimulation of cortical cells in the absence of an odor will then lead to excitation patterns of the GCs that are strongly correlated with the patterns excited by the training odor (Fig 4C and 4D). Previous experiments in which odor-evoked and context-evoked GC activation patterns were found to be correlated in different animals are suggestive of this outcome [26].

On a behavioral level the model makes specific predictions for the learning of odor discrimination or detection in cluttered environments. Recent experiments have shown that the detection of an odor in a go/no-go task is particularly difficult if that odor is masked or occluded by an odor [46]. Our model predicts that the performance in such a detection task would be enhanced if the animal is first familiarized with the occluding odor over an extended period of time [24]. The resulting restructuring of the bulbar network would lead to a reduction in the response to that familiar odor, partially unmasking the task-relevant odor. If, in addition, the occluding familiar odor has been associated with a non-olfactory context [26], the model predicts that the performance is further enhanced if the task is performed in that context, but not in a different, novel context (Fig 5A).

Even if the cluttered odor environment does not occlude the task-relevant odors, it is expected that the learning speed in an odor-discrimination task [35] is reduced by strong distracting odors. The model predicts that sufficient familiarization [24] with the distracting odors will reduce their uninformative contributions to the overall MC activation pattern. This is expected to increase the signal-to-noise ratio and with it the learning speed by reducing the contributions from the uninformative MCs to the variability of the read-out. If, in addition, the distracting, uninformative odors are associated with a context, the learning speed is predicted to increase further in the presence of that context (Fig 6B). If the cluttered environment makes the task too hard to learn for naive animals, our model suggests that prior familiarization with the distracting odors, preferably in a specific context, may enable the animals to master this difficult task.

## Discussion

Adult neurogenesis is a striking mechanism of structural plasticity that has the potential to rewire a network extensively. In mammals it arises predominantly in two brain areas. In the dentate gyrus it involves excitatory granule cells; their role in the network has been studied in detail [52, 53], also in terms of computational models [54]. In the olfactory system, where adult neurogenesis involves inhibitory rather than excitatory granule cells, there is also a substantial body of experimental work [20], but only few modeling studies are available [39, 55]. Here we have developed a computational model for the neurogenic evolution of the network connectivity with an emphasis on the possible role of the pervasive top-down projections from cortical areas. The model is based on a number of experimental observations: GC survival depends on GC activity [21, 38], GC activity can be induced in the absence of odor stimulation [26], and piriform cortex exhibits extensive recurrent excitation, which can support associational memory [30, 31]. The model captures qualitatively the experimentally observed perceptual learning afforded by neurogenesis [24] as well as the enhanced apoptosis of specific GCs and the reduced odor discriminability after the extinction of memories [25].

Without theoretical guidance, it is difficult in experiments to identify the functional structure of the cortico-bulbar connectivity, in particular, because odor representations in the olfactory system do not reflect detailed spatial maps like those of other sensory systems [56, 57]. An important contribution of the model is therefore its prediction that through the structural plasticity the network develops a structure that reflects the learned odors and provides enhanced inhibition that is specific to these odors. This inhibition is in part intra-bulbar and in part driven by top-down (cortical) inputs. The latter reflects the formation of a bidirectional connection between the bulbar and the cortical representation of the familiar odors, which allows the cortical cells associated with such an odor to inhibit specifically those MCs that are excited by that odor. This inhibition is mediated by GCs. For this connectivity to arise the reciprocal nature of the MC-GC synapses is essential. Our results therefore suggest that a key function of the reciprocity of these synapses may be to guide the wiring of the cortico-bulbar network connectivity. The predicted matching of the GC receptive fields for olfactory and for cortical input can be tested experimentally. Moreover, the model can guide future experiments aimed at elucidating the cortico-bulbar connectivity.

Functionally, the model predicts, in particular, that the learned connectivity improves the detection and discrimination of novel odors in cluttered environments, if the occluding or distracting odors are familiar. This is achieved by a reorganization of the cortical-bulbar network so as to enhance the inhibition of familiar odors. Processing of odors in cluttered environments can also be enhanced by adaptation of sensory neurons or adaptation further downstream [58]. For those mechanisms to be successful, the occluding/distracting stimuli need to be present before the novel stimulus. In contrast, the neurogenically formed structured network suppresses familiar distractors and occluders independent of their relative onset times, even if the novel odor precedes the occluding or distracting odor.

Moreover, the processing of cluttered environments can be enhanced by top-down input when the occluding or distracting odor activates cortical memory. This may indicate that a higher brain area has recognized parts of the odor scene and may allow something akin to the ‘explaining away’ of components of a complex odor mixture that is theoretically predicted for the optimal processing of stimuli [8–10, 16]. Recent work has identified networks that demix familiar odors employing approximate optimal Bayesian inference; the anatomical structure of these networks is very close to that emerging naturally in our neurogenic model [10, 11]. By providing a biophysically supported mechanism through which the system can learn the required network structure our model complements this abstract normative approach.

Going beyond the purely olfactory aspect, the top-down input could encode task-related expectations or contextual information originating from other sensory modalities. Thus, it may implement a predictive coding in the bulb that reflects the context or task at hand [59]. Our model demonstrates how such contextual information can enhance performance.

The formation of the bulbar and cortico-bulbar network via structural plasticity is a relatively slow process. However, our model predicts that the network structure emerging from it can be exploited by faster synaptic learning processes in cortex, which allow the system to switch relatively quickly between different discrimination tasks. This is reminiscent of the task-dependent switching of neuronal responses observed in V1 [7].

Focusing on the slow evolution of the network structure our model is intentionally minimal with respect to the dynamics of the individual neurons. We describe the neurons in terms of their firing rate and focus on neuronal steady-state activities. So far, the knowledge about the biophysical mechanisms controlling GC survival and their dependence on neuronal activity is not sufficient to guide the development of a detailed model of that process, which would, e.g., connect GC spiking or calcium-levels with GC survival [38, 60, 61].

To assess the discriminability of MC activity patterns we assumed that the firing rates result from an irregular firing in which the variance of the spike number is proportional, albeit not necessarily equal, to the mean spike number. Thus, we have not taken the widely observed rhythmic aspects of the bulbar activity into account [62], nor the possibility that animals may also be able to make use of spike-timing-, correlation-, or synchrony-information in odor processing [9, 63–66]. The modular network structure and the associated specific disynaptic inhibition of MCs by top-down inputs that are predicted by our model are likely to have impact also on spike timing and synchrony [67]. A study of the resulting dynamics and functional consequences is, however, beyond the scope of this paper and will be left to future work.

Not much is known experimentally about the parameter values for the model. There have been recent efforts to constrain various coupling strengths in bulbar-cortical rate models based on experimental measurements of firing rate correlations [68]. The two types of networks investigated there have, however, different types of connections than the network investigated here. For instance, in their models the bulbar inhibitory cells receive either input directly from the sensory neurons, which would identify them as periglomerular rather than granule cells (e.g. [69]), or only by the cortical cells but not from the mitral cells. The observed reciprocal nature of the synapses between GCs and MCs plays, however, a central role in our model. It is therefore not clear to what extent the results in [68] directly provide constraints for the strength of the connections included in our work. It would be of interest to apply their approach to the class of networks obtained here to obtain some additional guidance for the choice of parameters. We have checked that our key results are quite robust under parameter changes as long as the cortical network does not become bistable, i.e. as long as it does not sustain activity without any bulbar or contextual input.

We have used a minimal model to focus on the dominant aspects associated with neurogenic network restructuring. Thus, in the bulb we have omitted the odor processing performed in the glomerular layer, which may also contribute to pattern separation and normalization [70, 71]. Our cortical model aimed to capture only two aspects of cortical processing: the formation of memories and the association of odors with contexts, both through excitatory lateral connections with Hebbian plasticity and all-to-all recurrent inhibition. We have not included multiple layers of principal cells nor feedforward inhibition from the bulb [72].

The olfactory system most likely gains additional richness through the nonlinear response properties of the GCs, which include local dendritic depolarization that can provide reciprocal inhibition to the MCs even without spiking [73], as well as wide-spread dendritic activation associated with calcium- or sodium-spikes that may drive wide-spread lateral inhibition [74, 75]. Thus, it is likely that inhibition can operate in multiple regimes [76], which may further enhance the system’s ability to switch between different tasks.

Adult neurogenesis is not the only mechanism contributing to the plasticity of the olfactory bulb, which is, for instance, reflected in the temporal evolution of the receptive fields of adult-born GCs [77] and in changes in the connectivity between MCs and GCs that occur during learning [78]. Spike-timing dependent plasticity is known to be enhanced in the proximal synapses of young adult-born GCs [4, 79]. At the reciprocal synapses between MCs and GCs long-term depression [80, 81] and bidirectional plasticity driven by single bursts [82] have been observed. In addition, the reciprocal synapses exhibit substantial structural plasticity in the form of spine fluctuations in adult-born as well as neonatally born GCs [18, 19]. In computational models of the olfactory bulb without top-down inputs spine fluctuations and adult neurogenesis lead to very similar network connectivities [19, 39]. We therefore expect that a cortico-bulbar model based on spine fluctuations would lead to results that are very similar to the ones based on adult neurogenesis described here. How the two types of structural plasticity complement each other and how the sequential formation of proximal and apical synapses [47] affects the emerging connectivity are interesting questions, which are, however, beyond the scope of this paper.

## Methods

Our model consisted of three populations of neurons, mitral/tufted cells (*M*), granule cells (*G*), and ‘cortical’ cells (*C*). Although the differences between mitral and tufted cells in terms of their properties and function are becoming increasingly known [83–85], the model did not distinguish between them. The neuronal activities were described by the nonlinear rate equations

$$\begin{array}{ccc} \tau_{M}\frac{dM_{i}}{dt} & = & -M_{i}+S_{i}+M_{sp}-\\ & & -g\sum_{j}W_{ij}^{\left(MG\right)}{\left[G_{j}-G_{th}\right]}_{+}, \end{array}$$ (1)

$$\begin{array}{ccc} \tau_{G}\frac{dG_{i}}{dt} & = & -G_{i}+\sum_{j}W_{ij}^{\left(GM\right)}{\left[M_{j}\right]}_{+}+\\ & & +w_{GC}\sum_{j}W_{ij}^{\left(GC\right)}\sigma \left(C_{j}\right), \end{array}$$ (2)

$$\begin{array}{ccc} \tau_{C}\frac{dC_{i}}{dt} & = & -C_{i}+w_{CM}\sum_{j}W_{ij}^{\left(CM\right)}{\left[M_{j}\right]}_{+}+\\ & & +\sum_{j\neq i}(\alpha W_{ij}^{\left(CC\right)}-w_{inh})\sigma \left(C_{j}\right). \end{array}$$ (3)

The odor stimuli were given by *S*_*i*_ and the spontaneous MC activity by *M*_*sp*_. A minimal, rectifying nonlinearity was chosen for the MCs and GCs,

$$\begin{array}{c} {\left[M\right]}_{+}=\{\begin{array}{cc} M & \text{for}\;M>0\\ 0 & \text{for}\;M\leq 0 \end{array}. \end{array}$$

To allow for pattern completion in the cortical area the nonlinearity for the CCs was chosen to be sigmoidal

$$\begin{array}{c} \sigma \left(C\right)=\frac{C_{max}}{1+e^{-\gamma_{C}(C-C_{th})}}. \end{array}$$

The bulbar connectivity matrices satisfied

$$\begin{array}{c} W_{ij}^{\left(MG\right)}=W_{ji}^{\left(GM\right)}, \end{array}$$

reflecting the important reciprocity of the MC-GC synapses. The entries of $W_{ij}^{\left(GM\right)}$ were given by 0 or 1. Without loss of generality the strength of the excitatory synapses from the MCs to the GCs was set to 1, while the strength of the reciprocal inhibition was given by *g*.

The CCs received excitatory inputs of strength *w*_*CM*_ from the MCs via the connectivity matrix $W_{ij}^{\left(CM\right)}$, i.e. the entries of $W_{ij}^{\left(CM\right)}$ were 0 or 1. In all of our computations except for those investigating the impact of sparse cortical representations (cf. S6 Fig in Supporting Information) $W_{ij}^{\left(CM\right)}$ was taken to be the identity matrix. The excitatory cortical synapses were taken to be plastic. In all the simulations presented in this paper the cortical connectivity was learned at the beginning of the simulations using the Hebbian plasticity rule

$$\begin{array}{c} W_{ij}^{\left(CC\right)}\to W_{ij}^{\left(CC\right)}+\eta \{(\sigma (C_{i})-\Omega )\sigma (C_{j})-\kappa \} \end{array}$$ (4)

with hard limits given by $0\leq W_{ij}^{\left(CC\right)}\leq W_{max}^{\left(CC\right)}$. Here, the cortical activities *C*_*i*_ were given by the steady state reached for the current values of $W_{ij}^{\left(CC\right)}$. The learning rate was given by *η*, the threshold for potentiation by Ω. There was an overall slow decay of the weights given by *κ*. The parameter *α* in Eq (3) allowed a reduction of the recurrent excitation during learning to reduce interference with previously learned memories [86] by switching between *α*_*learn*_ and *α*_*recall*_. During the neurogenic network evolution the strengths of the recurrent cortical connections were held fixed. There was also all-to-all inhibition among the cortical cells with strength *w*_*inh*_.

The connectivities $W_{ij}^{\left(MM\right)}$ and $W_{ij}^{\left(MC\right)}$ of the effective disynaptic inhibition between MCs and of MCs by CCs, examples of which are shown in Fig 1B, are given by

$$\begin{array}{c} W_{ij}^{\left(MM\right)}=\sum_{k=1}^{N_{G}}W_{ik}^{\left(MG\right)}W_{kj}^{\left(GM\right)},\;W_{ij}^{\left(MC\right)}=\sum_{k=1}^{N_{G}}W_{ij}^{\left(MG\right)}W_{kj}^{\left(GC\right)}, \end{array}$$ (5)

where *N*_*G*_ is the number of GCs. Note that the diagonal elements of $W_{ij}^{\left(MM\right)}$ are given by the number of non-zero elements in the corresponding rows of $W_{ij}^{\left(MG\right)}$ and are typically quite large. In the connectivity figures we have therefore replaced these large values by 0 to reveal the structure of the remaining connections. In the computations these diagonal elements were, of course, not set to 0.

In each time step of the neurogenic network evolution $N_{new}^{\left(G\right)}$ new GCs were added to the network, each making $N_{conn}^{\left(MG\right)}$ randomly chosen connections with MCs and $N_{conn}^{\left(GC\right)}$ randomly chosen connections with CCs. Then the steady-state values of *M*, *G*, and *C* for each odor $S_{i}^{\left(\beta \right)}$, *β* = 1…*N*_*s*_, in the odor environment were determined by solving the evolution Eqs (1), (2) and (3) for the corresponding odor until a steady-state was reached. Since only the steady-state values were desired we set *τ*_*G*_ = 0, which allowed a drastic reduction of the number of differential equations that had to be solved. Then the resilience of each GC was determined as the sum of its thresholded activity over all *N*_*s*_ training stimuli,

$$\begin{array}{c} R_{i}=\sum_{\beta =1}^{N_{s}}{\left[G_{i}^{\left(\beta \right)}-G_{th}\right]}_{+}, \end{array}$$ (6)

and GCs were removed depending on their survival probability *P*_*i*_ given by (cf. Fig 2)

$$\begin{array}{c} P_{i}=\frac{1}{2}(\tanh(\gamma_{R}(R_{i}-R_{0}))+1). \end{array}$$ (7)

This completed the neurogenic time step. For all results, except in Fig 3, sufficiently many such steps were taken to reach a statistically steady state in the network connectivity and in the various quantities assessing the features of the system. By using sufficiently many GCs the network fluctuations in this steady state were kept small enough to allow reliable measurements of the various relevant quantities.

The number of GCs was not fixed in this neurogenic model; instead, it depended on the strength of the inputs $S_{i}^{\left(\beta \right)}$ and the strength *g* of the inhibitory connections. For strong inhibition *g* the MC activities were low, leading to low activities of the GCs and a correspondingly low survival probability. As a result, the number of GCs was low. Conversely, choosing weak inhibition *g* lead to a large number of GCs. When the number of GCs was low, adding and removing a single GC had a significant effect on the MC and GC activities, resulting in strong fluctuations in the output of the network. To balance computational effort with the need to keep the fluctuations sufficiently low, we therefore adjusted in the simulations the inhibitory strength *g* during the network evolution to keep the number of GCs within a predetermined target range centered at $N_{GC}^{\left(aim\right)}$. Only in Fig 3, where the focus was the temporal evolution of the network, we kept *g* fixed after *t* = 18, 750 before the memory was extinguished at *t* = 25, 000.

Each model odor stimulus was given by *N*_*h*_ combinations of Gaussian activity profiles of the form

$$\begin{array}{c} S_{i}=\sum_{k=1}^{N_{h}}A_{k}e^{-\frac{{\left(i-x_{k}\right)}^{2}}{w_{k}^{2}}}. \end{array}$$ (8)

Contextual inputs drove the corresponding contextual cortical cells *C*_*i*_ with a cell-independent amplitude *A*_*context*_. In total there were $N_{CC}^{\left(context\right)}$ CCs representing the contexts.

To assess the discriminability of two stimuli $S_{i}^{\left(1\right)}$ and $S_{i}^{\left(2\right)}$ we used the optimal Fisher discriminant *F*_*opt*_

$$\begin{array}{c} F_{opt}=\sum_{i=1}^{N_{MC}}\frac{{\left({\left[M_{i}^{\left(1\right)}\right]}_{+}-{\left[M_{i}^{\left(2\right)}\right]}_{+}\right)}^{2}}{{\left[M_{i}^{\left(1\right)}\right]}_{+}+{\left[M_{i}^{\left(2\right)}\right]}_{+}} \end{array}$$ (9)

and a non-optimal Fisher discriminant *F*_*nonopt*_, which was obtained as the mean of 5,000 Fisher discriminants *F*_*random*_,

$$\begin{array}{c} F_{nonopt}=\langle F_{random}\rangle , \end{array}$$

each of which was based on a random read-out of the MC activities,

$$\begin{array}{c} F_{random}=\frac{{\left(\sum_{i=1}^{N_{MC}}w_{i}^{\left(random\right)}({\left[M_{i}^{\left(1\right)}\right]}_{+}-{\left[M_{i}^{\left(2\right)}\right]}_{+})\right)}^{2}}{\sum_{i=1}^{N_{MC}}{\left(w_{i}^{\left(random\right)}\right)}^{2}({\left[M_{i}^{\left(1\right)}\right]}_{+}+{\left[M_{i}^{\left(2\right)}\right]}_{+})}. \end{array}$$ (10)

Here the weights $w_{i}^{\left(random\right)}$ were real numbers between -1 and +1 drawn from a uniform distribution. By including negative weights we allowed for the possibility that the hypothetical read-out cell could also receive disynaptic inhibition from the MCs.

In the simulation experiments presented in the body of this paper we used one set of parameters. We have, however, tested that our results are robust under parameter changes (cf. S5 Fig in Supporting Information). The parameters used here are as follows:

$$\begin{array}{c} \tau_{M}=1\;\tau_{G}=0\;\tau_{C}=1, \end{array}$$

$$\begin{array}{c} M_{sp}=0.2\;G_{th}=3\;w_{GC}=3, \end{array}$$

$$\begin{array}{c} \alpha_{learn}=0.05\;\alpha_{recall}=1, \end{array}$$

$$\begin{array}{c} \gamma_{C}=3\;C_{th}=0.2\;C_{max}=1\;w_{inh}=0.05, \end{array}$$

$$\begin{array}{c} \Omega =0.2\;\kappa =0.1\;\eta =10\;W_{max}^{\left(CC\right)}=0.06, \end{array}$$

$$\begin{array}{c} N_{conn}^{\left(MG\right)}=16\;N_{conn}^{\left(GP\right)}=2\;N_{new}^{\left(G\right)}=0.1\cdot N_{GC}^{\left(aim\right)}\;N_{CC}^{\left(context\right)}=48 \end{array}$$

$$\begin{array}{c} \gamma_{R}=5\;R_{0}=3 \end{array}$$

$$\begin{array}{c} N_{GC}^{\left(aim\right)}=\{\begin{array}{cc} 2,000 & \text{Fig}.1\text{,}\;\text{Fig}.3,\;\text{Figs}.5,6\\ 4,000 & \text{Fig}.4\\ 16,000 & \text{Fig}.7 \end{array} \end{array}$$

The parameter values for the training stimuli stimuli were

$$\begin{array}{c} A_{k}=2-M_{sp}\;w_{k}=12\;A_{context}=2. \end{array}$$ (11)

The same parameters were used for the probe stimuli in Figs 3 and 7. In Fig 5 the parameters of the target in the probe stimulus were

$$\begin{array}{c} A_{k}=0.72\;w_{k}=2, \end{array}$$

while in Fig 6 the parameters for the targets were

$$\begin{array}{c} A_{k}=1.08\;w_{k}=12. \end{array}$$

The parameters for the occluder and the distractor in Figs 5 and 6 were also given by Eq (11).

The Matlab code implementing this model is available from the ModelDB web site senselab.med.yale.edu/modeldb under access number 247188.

## Supporting information

S1 Fig Extinction of a memory. (PDF)

S2 Fig Top-down connections mediate activation by context. (PDF)

S3 Fig Natural stimuli. (PDF)

S4 Fig Addition and removal of GCs induced fluctuations in the connectivity. (PDF)

S5 Fig Robustness under parameter variation. (PDF)

S6 Fig Expansion into cortex and sparse cortical representation. (PDF)

S7 Fig Quantification of the change in network specificity with *w*_*GC*_. (PDF)

S8 Fig Role of top-down input in non-optimal detection in the presence of a distractor. (PDF)

S9 Fig Role of top-down input in optimal detection in the presence of an occluder. (PDF)

S10 Fig Context does not enhance discriminability of CC-activity patterns. (PDF)

S11 Fig Discrimination with reduced top-down weight. (PDF)
