# Supplementary material for: Top-down inputs drive neuronal network rewiring and context-enhanced sensory processing in olfaction
Source: PLoS Comput Biol. 2019 Jan 22;15(1):e1006611. doi: 10.1371/journal.pcbi.1006611 (PMC6358160; doi:10.1371/journal.pcbi.1006611)
Supplement: S1 Fig — (PDF) [file pcbi.1006611.s001.pdf]

Supporting information

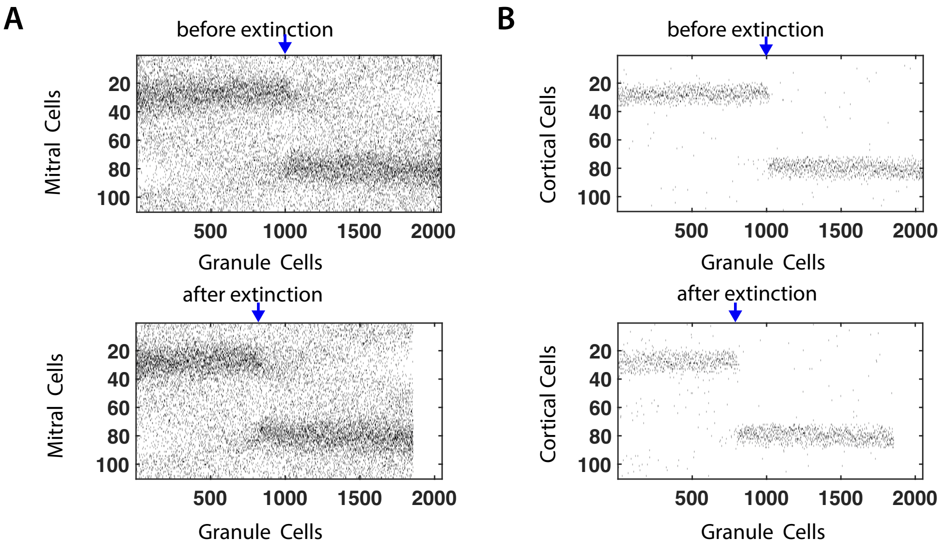

Fig S1. Extinction of a Memory.
Extinguishing the memory of odor pair 1 removed particularly GCs that had been associated with that odor pair (cf. Fig.3). (A): Connectivity between MCs and GCs before (top) and after (bottom) extinguishing the memory. The number of GCs activated by MCs with index ∼30 was reduced by the extinction (compare position of the arrows). (B): Connectivity between CCs and GCs before (top) and after (bottom) extinguishing the memory. The number of GCs excited by CCs with index ∼30 was reduced by the extinction (compare position of the arrows).
