# Supplementary material for: Top-down inputs drive neuronal network rewiring and context-enhanced sensory processing in olfaction
Source: PLoS Comput Biol. 2019 Jan 22;15(1):e1006611. doi: 10.1371/journal.pcbi.1006611 (PMC6358160; doi:10.1371/journal.pcbi.1006611)
Supplement: S2 Fig — (PDF) [file pcbi.1006611.s002.pdf]

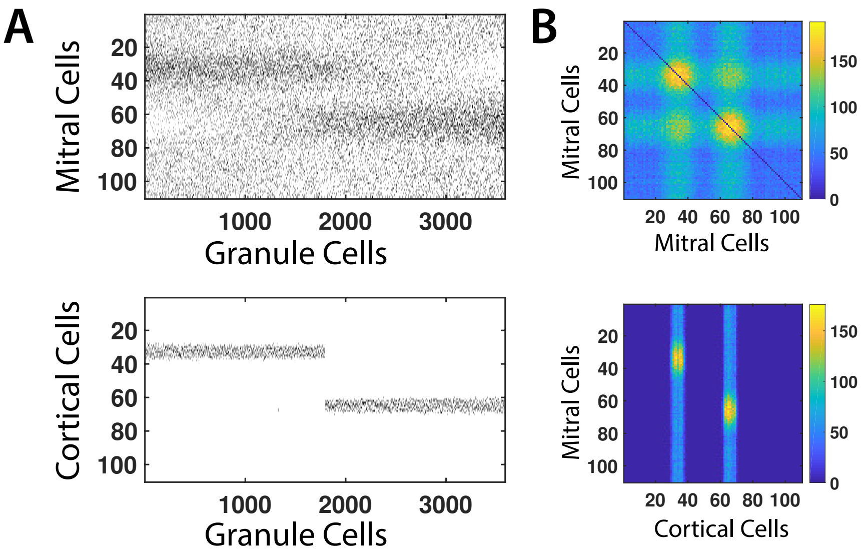

Fig S2. Top-down Connections Mediate Activation by Context.
Cortical cells with indices ∼30 were activated by context 1 via associational connections (cf. Fig.4B). Through the cortical projections they activated specifically GCs with index below 1805 ((A) bottom), which in turn inhibited predominantly MCs with index ∼30 via the reciprocal connections between MCs and GCs ((A) top). Analogously for context 2. Thus, the context excited approximately the same set of GCs as the odor associated with that context. Due to the reciprocal nature of the MC-GC synapses the context therefore disynaptically inhibited the MCs that would be excited by that odor ((B) bottom). These GCs also mediated mutual inhibition between the MCs ((B) top).
