# Supplementary material for: Top-down inputs drive neuronal network rewiring and context-enhanced sensory processing in olfaction
Source: PLoS Comput Biol. 2019 Jan 22;15(1):e1006611. doi: 10.1371/journal.pcbi.1006611 (PMC6358160; doi:10.1371/journal.pcbi.1006611)
Supplement: S3 Fig — (PDF) [file pcbi.1006611.s003.pdf]

While we used extremely simple stimuli to illustrate the connectivities emerging from the neurogenic network restructuring and the functionality of the resulting networks, our results are not limited to such stimuli. We illustrate this here for the case of context-enhanced odor detection using naturalistic stimuli based on glomerular activation patterns obtained by Johnson and Leon (e.g. [40]).

Fig.S3A shows the glomerular activation patterns for butyric acid, propionic acid, and dodecanal, down-sampled to reduce the computational effort. The network was trained on propionic acid and dodecanal. The one-dimensional representation of these odors is shown in Fig.S3B, where for visualization purposes the mitral cells have been ordered according to the difference in the glomerular activation by the two training odors. The training results in the network connectivities $W^{(MM)}$, $W^{(MC)}$, and $W^{(CC)}$ (for $w_{GC} = 3$), where $W^{(MC)}$ and $W^{(CC)}$ reflect the sparse activation of the CCs that was obtained for these parameters (Fig.S3C). Each of the two training odors was associated with a different context, as reflected in $W^{(CC)}$. As target odor we chose butyric acid in combination with the propionic acid as an occluding odor (Fig.S3D).

After learning, the network predominantly suppressed the occluding odor and made the target odor more easily detected, since the difference between the occluder and the target was not significantly reduced while the overall MC amplitude was substantially smaller (Fig.S3E for $w_{GC} = 3$). Without any context the inhibition was mostly intra-bulbar. In the presence of the context associated with the occluder ('correct context') the strong cortical contribution further increased the detectability. This was reflected in the enhanced value of the Fisher discriminant (Fig.S3E for three different values of the top-down connection strength, $w_{GC} = 2$, $w_{GC} = 3$, and $w_{GC} = 5$). In the presence of the context associated with dodecanal ('incorrect context') the cortically driven inhibition targeted the bulbar representation of dodecanal rather than that of the occluding proprionic acid and therefore did not enhance the detection of the target odor (Fig.S3E).

Even though the two training stimuli were very different, both drove a common set of MCs at much larger amplitudes than the simple Gaussian stimuli used in Fig.5. This made the learning of selective connectivities more difficult. Compared to the intra-bulbar connectivity obtained in the Gaussian case (Figs.5B,S4A), $W^{(MM)}$ had therefore significantly more connections between MCs that predominantly responded to propionic acid and MCs that responded strongly to dodecanal. Recall, that in Fig.S3 the MCs have been ordered according to the difference in their response to the two training odors; a highly selective connectivity would therefore have very few connections in the upper right or lower left quadrant (cf. the dashed squares in Fig.S4A). Nevertheless, the overall connectivity was selective enough for the two contexts to act very differently on the odor representations (Fig.S3E) with only the correct context enhancing the detectability of the target odor.

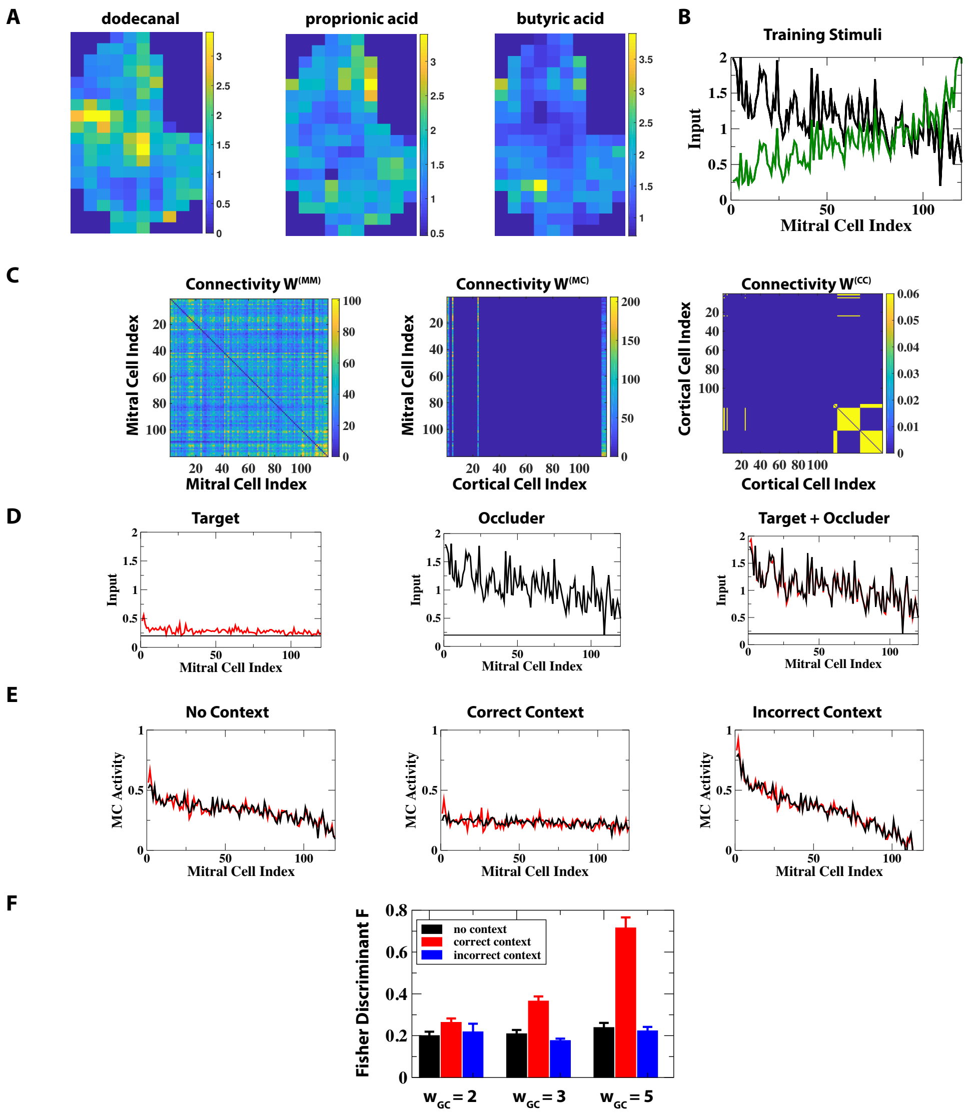

Fig S3. Natural Stimuli.
(A) Two-dimensional representations of the glomerular activation patterns for dodecanal, proprionic acid, and butyric acid used in the computations [40]. (B) One-dimensional representation of the inputs for the training stimuli dodecanal and proprionic acid with mitral cells ordered according to the difference between the two inputs. (C) Connectivities resulting from training on the stimuli shown in (B). The cortical connectivity $W^{(CC)}$ reflects the association of a different context with each of the two stimuli; cells receiving contextual inputs have indices beyond 110. (D) Stimuli: target (butyric acid) and occluder (proprionic acid). The right-most figure shows the target combined with the occluder (red) as well as the occluder alone (black). (E) MC activities for the target-occluder mixture (red line) and the occluder alone (black line), with and without context. The 'correct' context is associated with the occluder proprionic acid, while the 'incorrect' context is associated with dodecanal. (F) Fisher discriminant for the detection of the occluded target for three values of the top-down synaptic strength w_{GC}. With increasing top-down input the correct context enhances the detection of the target substantially. The parameter values used in these computations were the same as described in Methods except for $R_0 = 5$, $G_{th} = 5$, $N_{GC}^{(aim)} = 2,000$. We normalized the odor maps to the range $[0, 2 - M_{sp}]$. When comparing the target-occluder mixture with the occluder alone we adjusted the occluder amplitude to have a similar overall amplitude as the mixture.
