# Supplementary material for: Top-down inputs drive neuronal network rewiring and context-enhanced sensory processing in olfaction
Source: PLoS Comput Biol. 2019 Jan 22;15(1):e1006611. doi: 10.1371/journal.pcbi.1006611 (PMC6358160; doi:10.1371/journal.pcbi.1006611)
Supplement: S4 Fig — (PDF) [file pcbi.1006611.s004.pdf]

The addition and removal of GCs in our model is a stochastic process. The resulting temporal fluctuations in the connectivity during the resulting steady state are quantified in Fig.S4, which gives the mean and the standard deviation of the number of GC-mediated disynaptic connections between MCs ($W^{(MM)}$ in (A)) and from CCs to MCs ($W^{(MC)}$ in (B)). The size of the fluctuations increased with the strength g of the individual inhibitory synapses. The system's key feature is the emergence of a subnetwork structure that reflects the training stimuli, as indicated in Fig.1. As long as g was not too small, the fluctuations perturbed this structure only weakly. This is visually evident when comparing the size of the fluctuations relative to their mean in Fig.S4A,B. It is quantified in Fig.S4C, where the solid green and red bars labeled $W^{(MM)}$ give the total number of connections among MCs with similar receptive fields (indicated in Fig.S4A by green and red squares bounded by solid lines) and between MCs with different receptive fields (hashed bars and dashed black squares, respectively) and the error bars indicate the respective standard deviations of those sums. The bars labeled $W^{(MC)}$ in Fig.S4C give the corresponding results for the connections from CCs to MCs.

In all of our computations we chose the inhibitory strength g small enough to ensure that the results were robust with respect to these fluctuations. This is, for instance, apparent in Fig.3C,D,E, which shows the temporal fluctuations in the odor responses and the Fisher discriminant characterizing their discriminability. It is quantified by the error bars for the Fisher discriminant in Figs.5,6,7,S3.

Characterizing the connectivity obtained with the training stimuli of Fig.5, which consisted of two odors driving predominantly MCs with index near 26 and near 77, respectively.

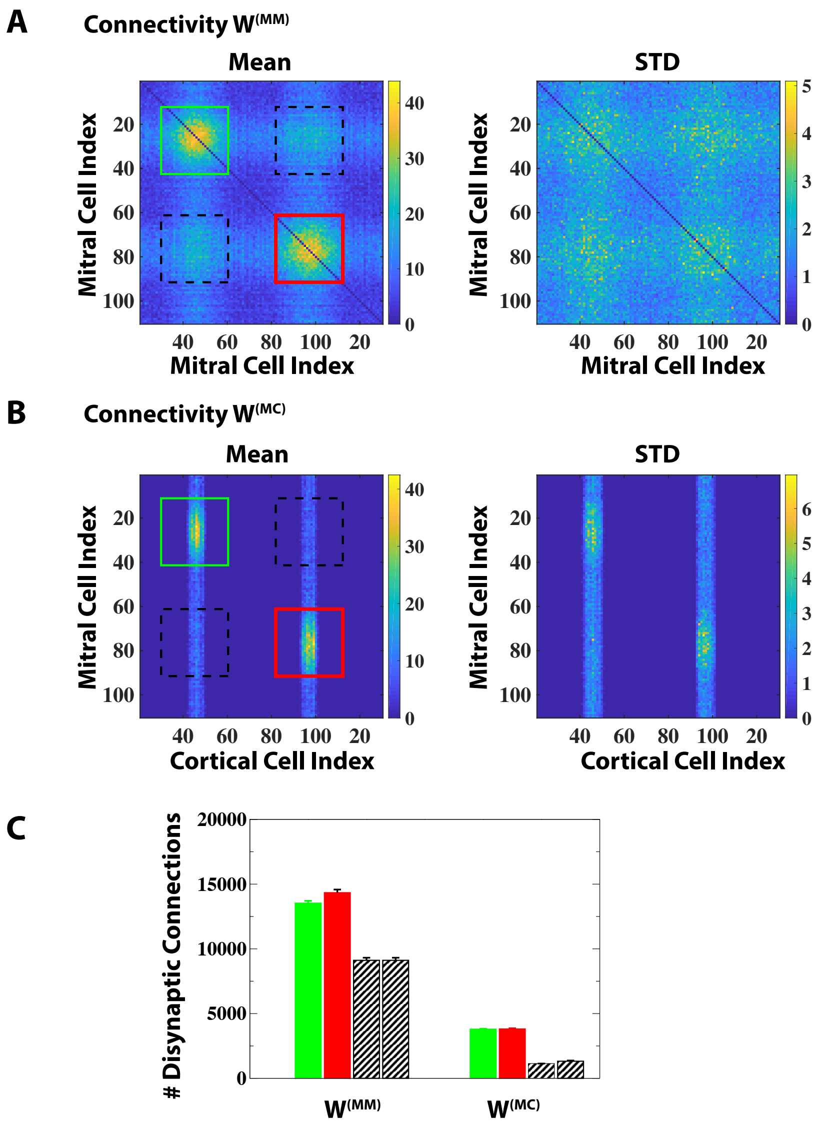

Fig S4. Addition and Removal of GCs Induced Fluctuations in the Connectivity.
(A) Left panel: mean number of GC-mediated inhibitory connections among MCs. The number of connections among MCs that respond to the same odor (marked by squares with solid lines) is substantially larger than between MCs with different receptive fields (squares with dashed lines). Right panel: standard deviation of the corresponding number of connections. It is much smaller than the mean.
(B) Analogous results for the top-down connectivity from CCs to MCs via GCs. Again, the size of the fluctuations in the number of connections is much smaller than their mean.
(C) Quantification of the number of connections among cells with the same receptive field (solid bars corresponding to the number of connections inside the solid squares in (A) and (B)) and between cells with different receptive fields (hashed bars corresponding to the squares with dashed lines). The standard deviation in the number of connections is indicated by the error bars. Parameters and stimuli were as in Fig.5.
