# Supplementary material for: Top-down inputs drive neuronal network rewiring and context-enhanced sensory processing in olfaction
Source: PLoS Comput Biol. 2019 Jan 22;15(1):e1006611. doi: 10.1371/journal.pcbi.1006611 (PMC6358160; doi:10.1371/journal.pcbi.1006611)
Supplement: S5 Fig — (PDF) [file pcbi.1006611.s005.pdf]

In order to demonstrate that the *same* system can learn quite different tasks - dealing with occluders, distractors, and switching between tasks - all the computations in Figs.5,6,7 have been performed for the same parameter values. Here we illustrate that the parameters do not have to be tuned very finely to obtain these results. Fig.S5 shows the key results of optimal detection, nonoptimal discrimination, and fast learning for 18 runs for which all parameters are drawn from a uniform distribution of width ±15% around the respective values in Figs.5,6,7. In most cases the networks perform quite well.

For some parameters - not included in Fig.S5 - the cortical network became bistable due to excessive lateral excitation or insufficient inhibition and no useful network connectivity emerged. In that situation CC-activity was sustained purely by associative inputs without any input from the MCs. As a result, even if the inhibition was so strong that all MCs were silenced, GCs survived driven by the CCs; in fact, their number kept increasing indefinitely. This was the case in 5 of 100 test runs.

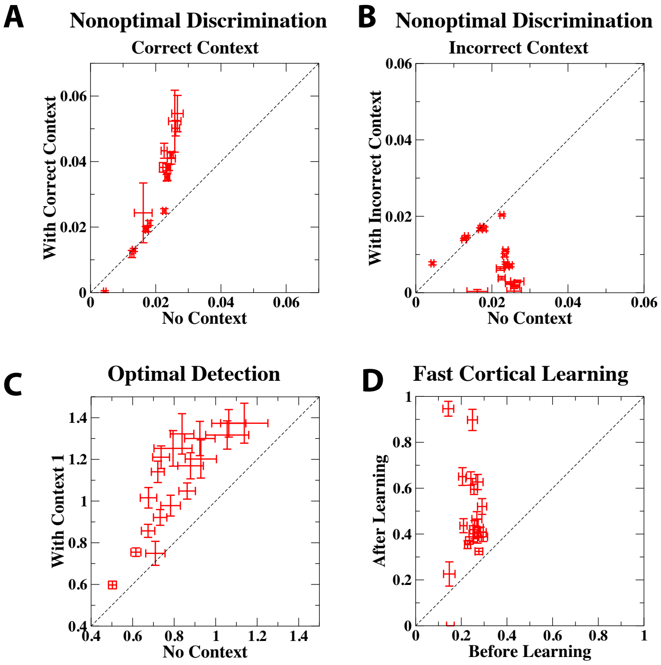

Fig S5. Robustness under Parameter Variation.
To confirm the robustness of the results shown in Figs.5,6,7 we repeated these computations 18 times with all parameters chosen randomly from a uniform distribution of width ±15% around their original values. While the performance depended on the specific parameter choice, only in a few cases did the network learn the task only pooly. (A) In the presence of the context associated with the distractor the two similar target odors were discriminated significantly better than without that context (cf. Fig.6), which was due to a reduction in the MC-activation by the distractor. (B) In contrast, in the presence of the incorrect context the odors were only poorly discriminated, since the cortically driven inhibition suppressed the target odors instead of the distractor (cf. Fig.6). (C) Context suppressed the occluding odor (cf. Fig.5) and enhanced the detection of the weak target odor. (D) After the cortical associative connections established connections between the two mixture components the two similar mixtures were significantly better discriminated (cf. Fig.7).
