# Supplementary material for: Top-down inputs drive neuronal network rewiring and context-enhanced sensory processing in olfaction
Source: PLoS Comput Biol. 2019 Jan 22;15(1):e1006611. doi: 10.1371/journal.pcbi.1006611 (PMC6358160; doi:10.1371/journal.pcbi.1006611)
Supplement: S6 Fig — (PDF) [file pcbi.1006611.s006.pdf]

In contrast to the 1:1 mapping of MCs to CCs used in our other computations, the mammalian bulb projects to olfactory cortex in an expansive manner, i.e. each MC projects to multiple CCs. These projections do not show any chemotopy but appear to be random. We therefore tested our model also with each CC receiving inputs from 2 randomly chosen MCs and allowing for a 6 times larger number of CCs. This increased the computational effort significantly, since each CC satisfied a nonlinear differential equation. An example of such a random projection is shown in Fig.S6B, where the CCs have been ordered according to their receptive field. Through the activity-dependent survival of the GCs the network developed the top-down connectivity shown in Fig.S6C, which together with the connectivities $W^{(MM)}$, $W^{(CM)}$, and $W^{(CC)}$ implied a subnetwork structure as sketched in Fig.1C. This allowed the CCs to inhibit disynaptically predominantly the MCs whose receptive field matched that of the CCs. As a result, the detection of an occluded stimulus (Fig.S6E, cf. Fig.5E) was enhanced by a context associated with the familiar occluder (Fig.S6G) in agreement with our results in Fig.5.

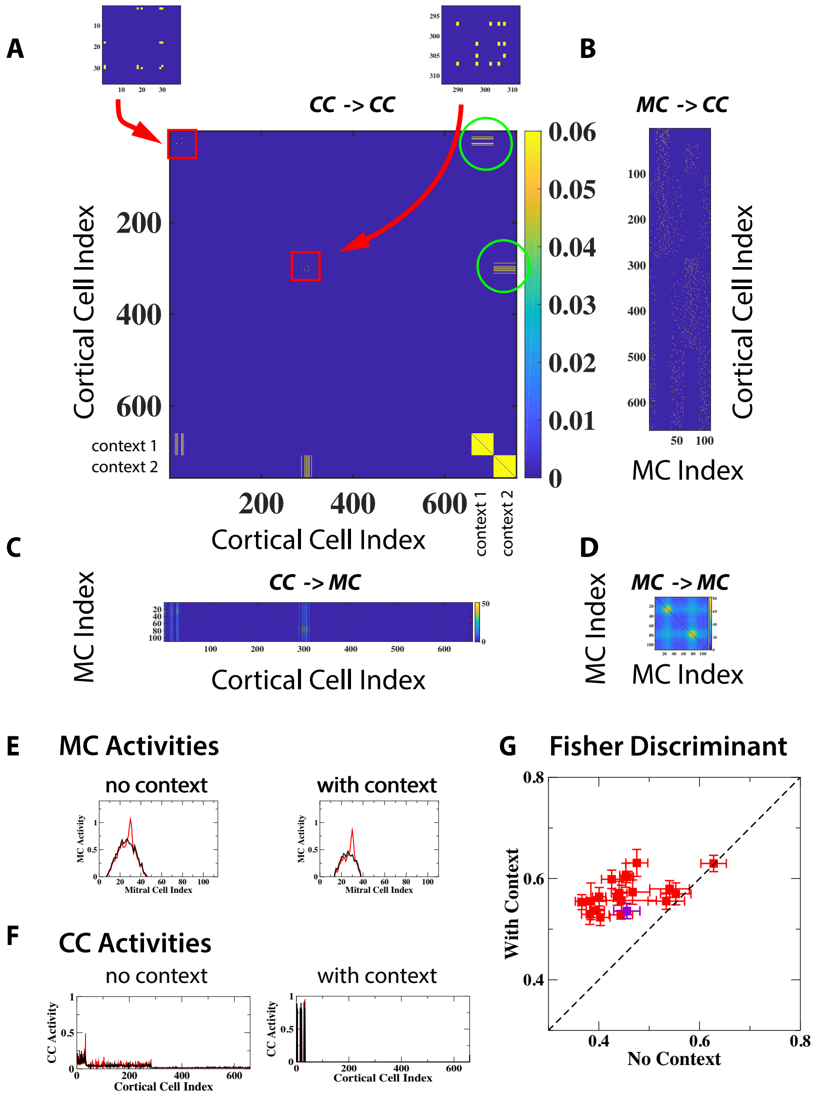

Fig S6. Expansion into Cortex and Sparse Cortical Representation. The random projection from the bulb to cortex shown in (B) led through Hebbian learning of the stimuli shown in Fig.5A to the very sparse associational connectivity $W^{(CC)}$ shown in (A). It includes the associational connections between cells driven by MCs and cells driven by context (marked by green circles). The 2 insets show enlargements of the associational connections between CCs driven by MCs (inside the red squares). Through the neurogenic network evolution the GCs mediated disynaptic inhibition of the MCs by CCs with a connectivity shown in (C). (D) The bulbar connectivity $W^{(MM)}$ was very similar to that obtained with a 1:1 mapping from the bulb to the cortex, as were the MC activity patterns induced by the occluded target and by the occluder alone (red and black lines, respectively in (E)). (F) Reflecting the sparse projection to the cortex, the cortical activity patterns were also very sparse. We tested the performance for 20 different randomly chosen projections $W^{(CM)}$ from the bulb to cortex, each of which led to different connectivities $W^{(MM)}$, $W^{(MC)}$, and $W^{(CC)}$. A sample $W^{(CM)}$ is shown in (B). Despite the sparseness of the cortical activity patterns the context associated with the occluder significantly enhanced the detection of the occluded target odor in most cases as shown by the larger Fisher discriminant obtained in the presence of the context than without that context ((G)). The purple symbol indicates the result obtained for the connectivities shown in (A, B, C, D).
Parameters were as described in *Methods* except for $N_{GC}^{(aim)} = 1,000$, $A_{context} = 3$, $N_{CC}^{(context)} = 96$, $w_{CM} = 0.65$, $\Omega = 0.1$, $\kappa = 0.07$.
