# Supplementary material for: Top-down inputs drive neuronal network rewiring and context-enhanced sensory processing in olfaction
Source: PLoS Comput Biol. 2019 Jan 22;15(1):e1006611. doi: 10.1371/journal.pcbi.1006611 (PMC6358160; doi:10.1371/journal.pcbi.1006611)
Supplement: S7 Fig — (PDF) [file pcbi.1006611.s007.pdf]

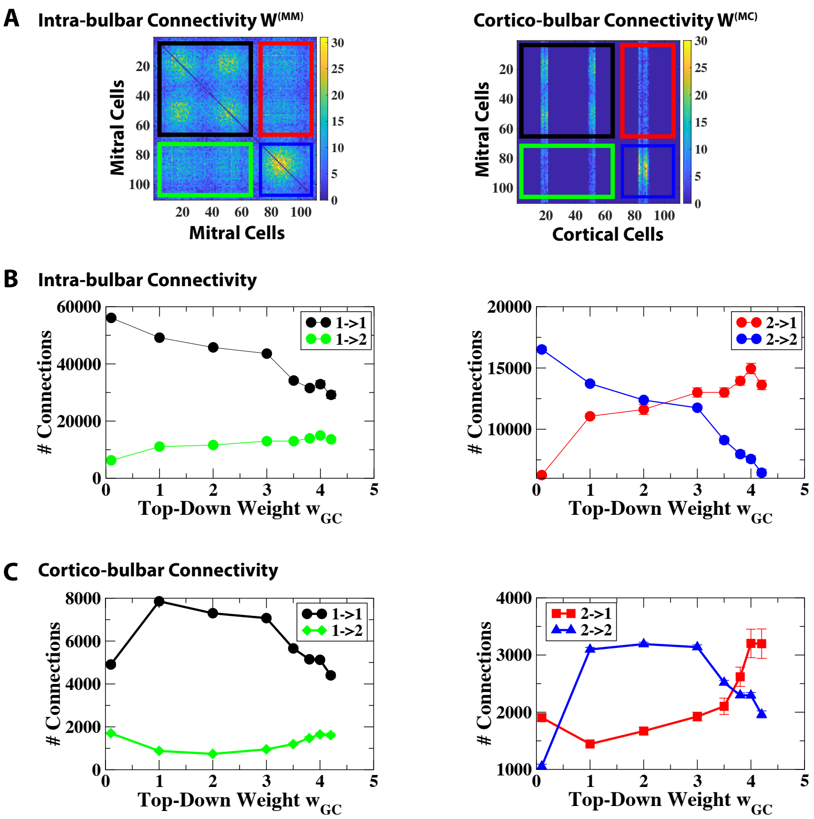

Fig S7. Quantification of the Change in Network Specificity with w_{GC}. (A) Connectivities $W^{(MM)}$ and $W^{(MC)}$ from Fig.8 for $w_{GC} = 3$. The black and blue squares in the left panel denote the connections that mediate mutual inhibition of MCs that are activated by the same training odor (odor 1 and 2, respectively). The red and green rectangles indicate connections among MCs that are activated by different odors. The right panel indicates the analogous connections from CCs to MCs. (B) The circles give the sum of all connections inside the rectangles with the corresponding color in the left panel of (A). With increasing w_{GC} the number of disynaptic inhibitory connections among the MCs driven by training stimulus 1 (black square in (A)) and among the MCs driven by training stimulus 2 (blue square in (A)) decreased (black and blue lines). The cross-inhibition of MCs by MCs driven by the other training stimulus, however, increased (green and red lines, corresponding squares in (A)). (C) Analogously to (B), the inhibition of MCs by CCs coding for the same stimulus decreased for large w_{GC} (black and blue lines), while that of MCs by CCs coding for the other stimulus increased (green and red lines). Thus, while the inhibition of the distractor by the context associated with it decreased with increasing w_{GC}, the inhibition of MCs driven by training stimulus 1 increased. This enhanced inhibition also suppressed the target stimulus, compromising its detection, as shown in Fig.8 and Fig.S8.
