# Supplementary material for: Top-down inputs drive neuronal network rewiring and context-enhanced sensory processing in olfaction
Source: PLoS Comput Biol. 2019 Jan 22;15(1):e1006611. doi: 10.1371/journal.pcbi.1006611 (PMC6358160; doi:10.1371/journal.pcbi.1006611)
Supplement: S8 Fig — (PDF) [file pcbi.1006611.s008.pdf]

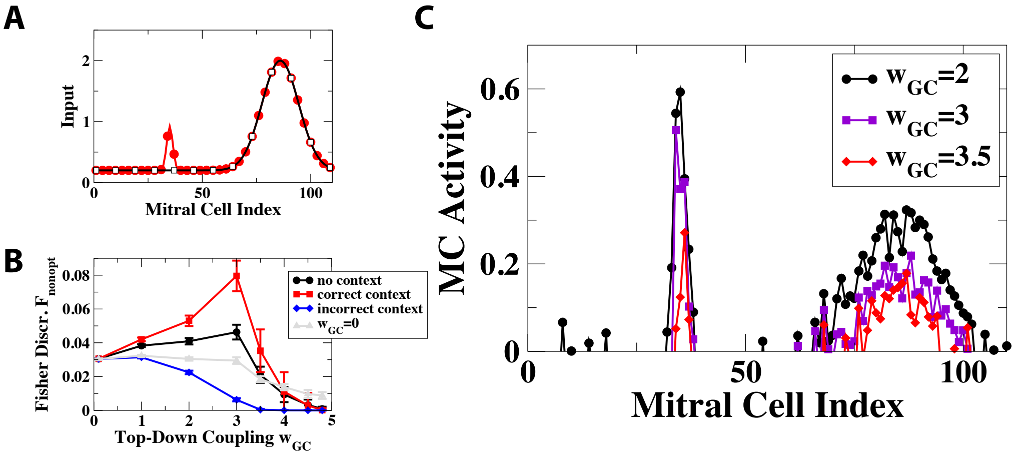

Fig S8. Role of Top-Down Input in Non-Optimal Detection in the Presence of a Distractor.

The task was to detect a weak target odor in the presence of a strong distractor. (A) Same stimuli as in Fig.8D (bottom panel). Red line: target with distractor, black line: distractor alone. (B) Dependence of $\mathcal{F}_{nonopt}$ on weight w_{GC} of the top-down connections (data of Fig.8E repeated for clarity). (C) MC-activities in response to the target in the presence of the distractor for three values of the top-down weight w_{GC}. To improve the detection of the weak target odor (MCs with index near 35) without optimizing the read-out weights the MC-activity driven by the distractor (MCs with index near 87) should be reduced more than the response to the target. This was the case for $w_{GC} = 2$ and even more so for $w_{GC} = 3$. For $w_{GC} = 3.5$, however, the connectivity lacked the selectivity needed to deliver specific inhibition (cf. Fig.S7) and the MC-activities driven by the target were substantially reduced, without a strong reduction in the activation by the distractor. As a consequence $\mathcal{F}_{nonopt}$ dropped substantially from $w_{GC} = 3$ to $w_{GC} = 3.5$.
