# Supplementary material for: Top-down inputs drive neuronal network rewiring and context-enhanced sensory processing in olfaction
Source: PLoS Comput Biol. 2019 Jan 22;15(1):e1006611. doi: 10.1371/journal.pcbi.1006611 (PMC6358160; doi:10.1371/journal.pcbi.1006611)
Supplement: S9 Fig — (PDF) [file pcbi.1006611.s009.pdf]

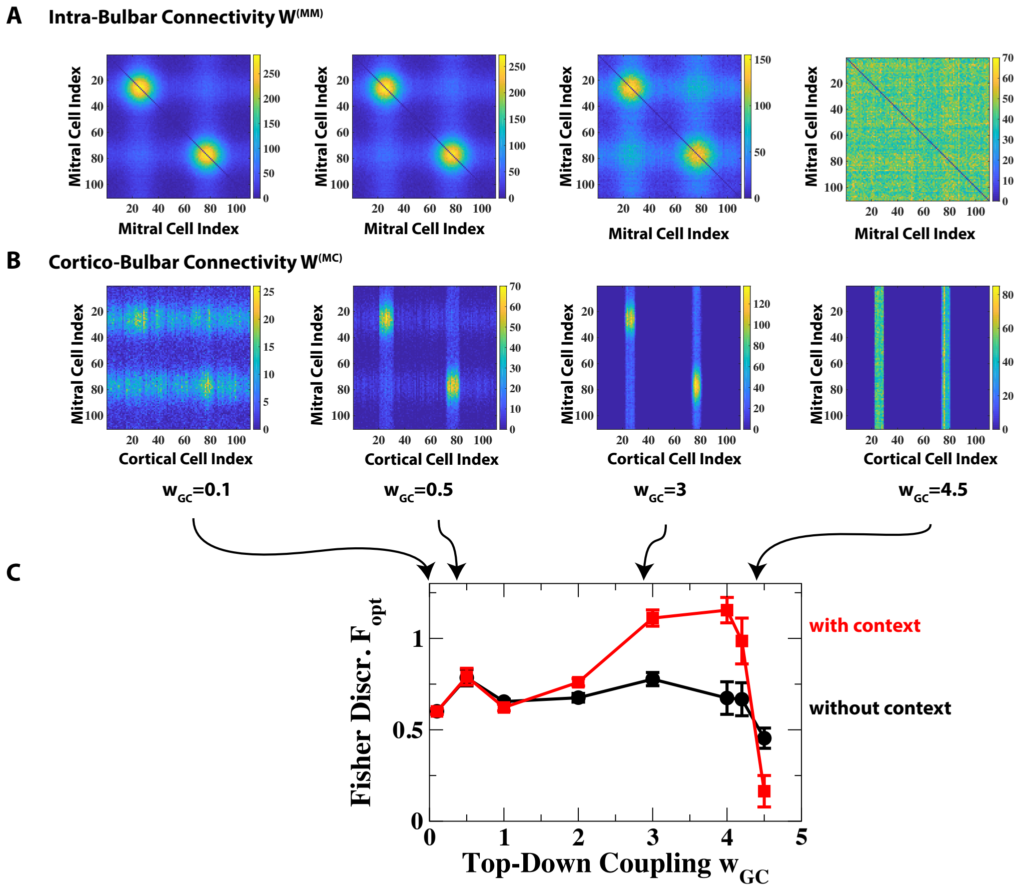


**Fig S9. Role of Top-Down Input in Optimal Detection in the Presence of an Occluder.**
The training stimuli were as in Fig.5A. (A) As the top-down weight $w_{GC}$ was increased the intra-bulbar connectivity became less selective (cf.Fig.8). (B) For small $w_{GC}$ almost all CCs had disynaptic inhibitory projections to MCs that responded to the training stimuli. For large $w_{GC}$ only CCs driven by the training stimuli projected to the bulb, but their disynaptic inhibition was unspecific. (C) Context enhanced the detection of the stimulus for an intermediate range in $w_{GC}$ in which both the intra-bulbar and the cortico-bulbar connectivity were selective.
