# Supplementary material for: Top-down inputs drive neuronal network rewiring and context-enhanced sensory processing in olfaction
Source: PLoS Comput Biol. 2019 Jan 22;15(1):e1006611. doi: 10.1371/journal.pcbi.1006611 (PMC6358160; doi:10.1371/journal.pcbi.1006611)
Supplement: S10 Fig — (PDF) [file pcbi.1006611.s010.pdf]

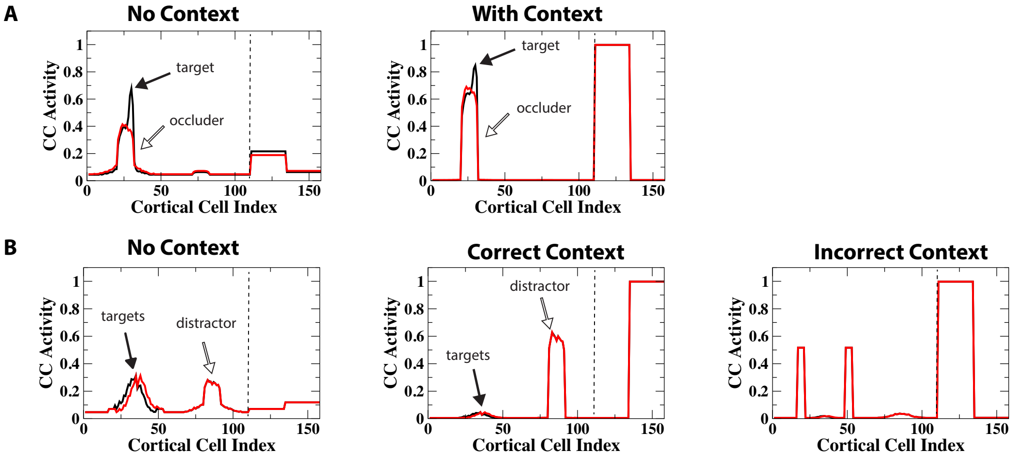


**Fig S10. Context Does Not Enhance Discriminability of CC-Activity Patterns**

(A) Cortical activity corresponding to Fig.5. Left panel: Without the input to the contextual CCs (to the right of the dashed line) the peak corresponding to the target odor is quite clearly discriminable on top of the occluder. Through the learned associational connections the CCs receiving odor input also drive the contextual CCs associated with the occluder. Right panel: In the presence of the context the contextual CCs strongly drive the CCs encoding the occluder, independent of the presence of the target. This reduces the detectability of the target in the presence of the occluder in terms of the CC-activity. This is in contrast to the enhanced discriminability of the MC-patterns in the presence of the context shown in Fig.5.
(B) Cortical activity corresponding to Fig.6. Left panel: Without any context the CC-pattern reflects both the target odors and the distractor. Middle panel: In the presence of the correct context the activity of the CCs encoding the distractor is enhanced while the global intra-cortical inhibition suppresses both target odors. Right panel: The incorrect context drives CCs that have indices close to those of the target odors. The global inhibition suppresses the CCs encoding the targets. Thus, both contexts strongly reduce the discriminability of the targets in terms of the CC-activities. In terms of the MC-patterns, however, the discriminability of the two target odors in the presence of the distractor is significantly enhanced by the correct context (Fig.6).
