# Supplementary material for: Top-down inputs drive neuronal network rewiring and context-enhanced sensory processing in olfaction
Source: PLoS Comput Biol. 2019 Jan 22;15(1):e1006611. doi: 10.1371/journal.pcbi.1006611 (PMC6358160; doi:10.1371/journal.pcbi.1006611)
Supplement: S11 Fig — (PDF) [file pcbi.1006611.s011.pdf]

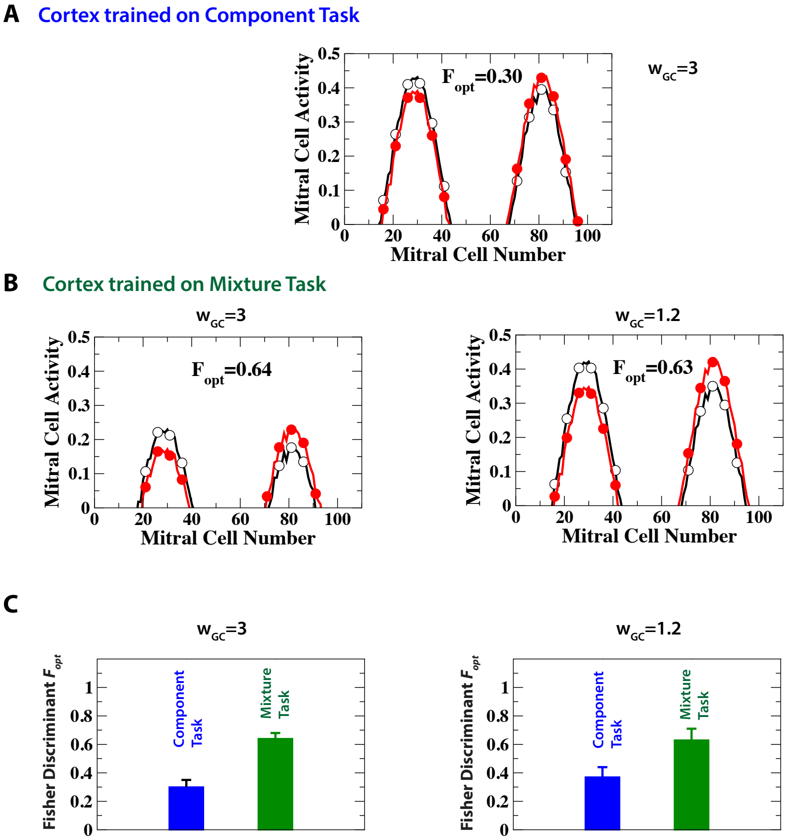

Fig S11. Discrimination with Reduced Top-down Weight
(A) MC-patterns for the mixture probe stimuli with cortex trained on the component task for $w_{GC} = 3$ (same data as in Fig.7). (B) MC-patterns for the mixture probe stimuli with cortex trained on the mixture task (cf. Fig.7E). Left panel: $w_{GC} = 3$, same data as in Fig.7. Since the mixture training activates many more associative connections than the component training, the top-down input induces substantially more inhibition in the MCs than for the cortex trained on the component task. The MC-patterns are therefore substantially weaker. To show that it was not simply the overall enhanced reduction that was responsible for the improved discrimination of the probe stimuli, the right panel shows the MC-patterns for the same connectivities, but with a reduced top-down weight (w_{GC}=1.2), chosen such that the overall amplitudes of the MC activities were comparable to those in (A). Despite the reduced value of w_{GC}, these MC-patterns were significantly more discriminable than the patterns in (A). (C) The Fisher discriminant quantifies that even with reduced top-down weight w_{GC} training the cortical network to the mixture task significantly enhanced the discriminability of the mixture despite the unchanged bulbar connectivity.
